# Integrated molecular characterization reveals potential therapeutic strategies for pulmonary sarcomatoid carcinoma

**DOI:** 10.1038/s41467-020-18702-3

**Published:** 2020-09-28

**Authors:** Zhenlin Yang, Jiachen Xu, Lin Li, Renda Li, Yalong Wang, Yanhua Tian, Wei Guo, Zhijie Wang, Fengwei Tan, Jianming Ying, Yuchen Jiao, Shugeng Gao, Jie Wang, Yibo Gao, Jie He

**Affiliations:** 1 Department of Thoracic Surgery, National Cancer Center/National Clinical Research Center for Cancer/Cancer Hospital, Chinese Academy of Medical Sciences and Peking Union Medical College, Beijing, P.R. China; 2 Department of Medical Oncology, National Cancer Center/National Clinical Research Center for Cancer/Cancer Hospital, Chinese Academy of Medical Sciences and Peking Union Medical College, Beijing, P.R. China; 3 Department of Pathology, National Cancer Center/National Clinical Research Center for Cancer/Cancer Hospital, Chinese Academy of Medical Sciences and Peking Union Medical College, Beijing, P.R. China; 4 State Key Lab of Molecular Oncology, National Cancer Center/National Clinical Research Center for Cancer/Cancer Hospital, Chinese Academy of Medical Sciences and Peking Union Medical College, Beijing, P.R. China

**Keywords:** Cancer genomics, Cancer genomics, Lung cancer, Lung cancer

## Abstract

Pulmonary sarcomatoid carcinoma (PSC) is a rare subtype of lung cancer with poor prognosis. Here, we perform multi-omics analysis of 56 PSC samples, 14 of which are microdissected to analyze intratumoral heterogeneity. We report the mutational landscape of PSC. The epithelial and sarcomatoid components share numerous genomic alterations, indicating a common progenitor. We find that epithelial-mesenchymal transition (EMT) plays important roles in the carcinogenesis of PSC. The pan-cancer analysis reveals high tumor mutation burden and leukocyte fraction of PSC. Integrated molecular classification shows three subgroups with distinct biology, prognosis and potential therapeutic strategies. Actionable mutations are enriched in C1 and C2, patients in C3 have a significantly longer overall survival, and C1 and C2 exhibit T-cell inflamed microenvironments. The three subgroups show molecular similarities to specific subtypes of conventional lung cancer. In conclusion, our study reveals the molecular characteristics and provides entry points for the treatment of PSC.

## Abstract (web summary)

Pulmonary sarcomatoid carcinoma (PSC) is a rare subtype of lung cancer with poor prognosis. Here the authors performed multi-omics analysis of human samples to investigate the mutational landscape of PSC and show three subgroups of PSC with distinct biology, prognosis and potential therapeutic strategies.

## Introduction

Pulmonary sarcomatoid carcinoma (PSC) is a highly aggressive and poorly differentiated type of lung cancer, accounting for ~0.4% of all lung cancers, with five subtypes: pleomorphic, spindle cell, giant cell carcinoma, carcinosarcoma, and pulmonary blastoma^1,2^. Patients with this rare tumor often have poor prognosis and are resistant to traditional platinum-based chemotherapy^3^.

Targeted and immune therapies have greatly advanced the treatment of conventional non-small cell lung cancer (NSCLC) and improved patient outcomes^4–8^, providing new opportunities for the therapeutic strategies of PSC. Targetable mutations in *MET* causing exon 14 skipping have been identified in PSC, providing a new treatment option, although present in a minority of cases^9^. In addition, previous studies have reported high programmed death ligand 1 (PD-L1) expression in PSC^10–12^, making immunotherapy a promising choice. However, because of its rarity, previous molecular data on PSC are limited and mostly based on the targeted sequencing of DNA. Comprehensive molecular profiling is needed to identify more potential therapeutic targets and evaluate the tumor microenvironment (TME) and immunogenicity to provide clues for the future prospective of immunotherapy in PSC.

Furthermore, one of the intriguing characteristics of PSC is that the tumor cells can mimic sarcoma-like elements (i.e., spindle cells, giant cells or true sarcomatous transformation), and the epithelial and sarcomatoid components can simultaneously exist in one tumor with different proportions and morphologies, exhibiting high intra- and intertumoral heterogeneity^2^. As a result, seeking the relationship between the two components and performing further molecular classification are important to deepen our understanding of the biology of PSC, predict prognosis, and guide treatment in clinical practice.

In the current study, we perform multi-omics analysis of 56 PSC samples, 14 of which are microdissected to study intratumoral heterogeneity. We report the mutational landscape and the high tumor mutation burden (TMB) and leukocyte fraction (LF) of PSC. The epithelial and sarcomatoid components share numerous genomic alterations, indicating a common progenitor, and epithelial–mesenchymal transition (EMT) is found to play an important role in the carcinogenesis, which may be regulated by DNA methylation dynamics. Integrated molecular classification shows three subgroups with distinct biology, prognosis, and potential therapeutic strategies, and the subgroups exhibit molecular similarities to subtypes of conventional lung cancer. In conclusion, our study provides insights into the molecular characteristics, carcinogenesis, and classification of PSC, thus bringing entry points for treatment of this rare malignancy.

## Results

### Samples and clinical data

The primary tumor samples and paired normal lung tissues from 56 patients who underwent surgery for PSC were collected. We microdissected 14 of the tumor samples to separate the sarcomatoid and epithelial component in each tumor. The sarcomatoid component included spindle cells, giant cells, or a mixture of both, whereas the epithelial component included squamous cell carcinoma (SCC) or adenocarcinoma (ADC) in seven cases each (Supplementary Data 1). Hematoxylin and eosin (H&E) staining images of the epithelial and sarcomatoid components of the 14 cases were presented in Supplementary Fig. 1. The average tumor purity for all the tumor samples was 85%.

The median age of the patients enrolled was 62.5 years (range, 45–78 years). Forty-two (75.0%) of the patients were male, 39 (69.6%) were smokers, and the percentage of males and smokers was significantly higher in the seven cases with SCC components than in those with ADC components (gender, *P* = 0.005; smoking status, *P* = 0.005, Fisher’s exact test). More than half of the patients were diagnosed at stage III or IV. The median follow-up time for this cohort was 19.5 months and 64.3% of the patients died during follow-up. Patients with advanced T stage (T3–T4), lymph node metastasis and advanced TNM stage (III–IV) exhibited a significantly worse prognosis (T stage, *P* = 0.013; lymph node metastasis, *P* = 0.018; TNM stage, *P* = 9.19 × 10^−5^, log-rank test). The clinical data are summarized in Table 1 and Supplementary Data 1.

**Table 1:** Clinicopathological characteristics of the study cohort.

	No. of patients (%)
*Age (years)*	
Median (range)	62.5 (45–78)
*Gender*	
Male	42 (75.0%)
Female	14 (25.0%)
*Smoking status*	
Smoker	39 (69.6%)
Non-smoker	17 (30.4%)
*Tumor family history*	
Yes	16 (28.6%)
No	40 (71.4%)
*T stage*	
T1	3 (5.4%)
T2	22 (39.3%)
T3	17 (30.4%)
T4	14 (25.0%)
*N stage*	
N0	32 (57.1%)
N1-2	24 (42.9%)
*M stage*	
M0	52 (92.9%)
M1	4 (7.1%)
*TNM stage*	
I	8 (14.3%)
II	15 (26.8%)
III	29 (51.8%)
IV	4 (7.1%)
*Histological subtype*	
Pleomorphic carcinoma	44 (78.6%)
Spindle cell carcinoma	9 (16.1%)
Carcinosarcoma	2 (3.6%)
Pulmonary blastoma	1 (1.8%)

### Genomic alterations

We conducted whole exome sequencing (WES) on DNA samples collected from 56 PSC patients to assess genomic alterations globally. The average sequencing depth was 271× for tumor samples and 145× for normal lung samples. Information on the sequencing quality of all the samples was provided in Supplementary Data 2. We identified an exonic mutation rate of 7.1 mutations per megabase on average. The mean mutation load for smokers and nonsmokers was 9.1 and 2.4 per megabase and 348 and 91 in total, respectively. The mutation load was significantly higher in smokers (*P* = 2.78 × 10^−6^, Wilcoxon rank-sum test) (Supplementary Fig. 2A), and there was a significant correlation between TMB and pack years of smoking among smokers (*ρ* = 0.417, *P* = 0.008, Spearman’s correlation) (Supplementary Fig. 2B).

**Fig. 1:**
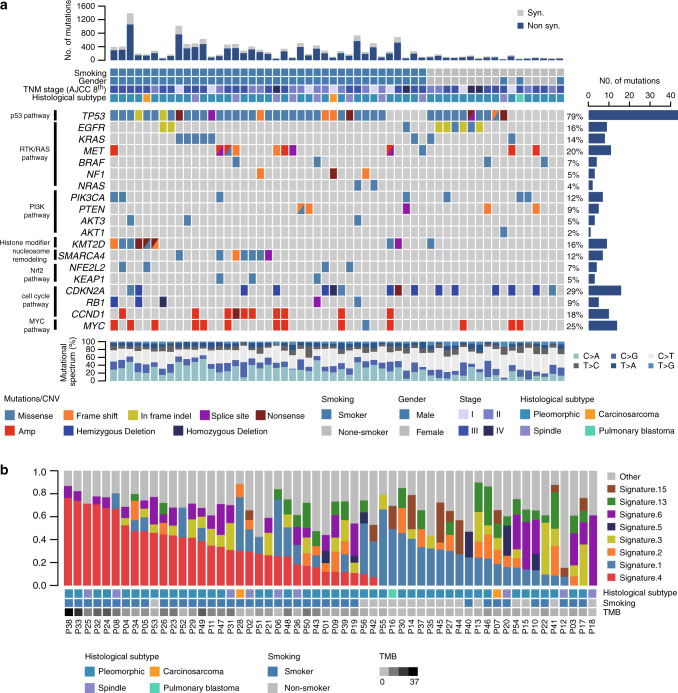
Genomic alterations in pulmonary sarcomatoid carcinoma (PSC). **a** Spectrum of the key molecular alterations in PSC. The genomic alterations of smokers and nonsmokers are demonstrated in the left and right side, respectively. The overall number of somatic mutations and clinicopathological data are shown at the top. The type of base-pair substitutions of each sample are displayed in the bottom panel. **b** Mutational signatures in our cohort (*n* = 56). Samples, annotated for the histological subtype, smoking status and tumor mutation burden, are ordered by the contribution of signature 4. Source data are provided as a Source data file.

The somatic mutations detected by WES are listed in Supplementary Data 3. Combined analysis using four software packages (MutSigCV, MuSiC, OncodriveCLUST, and Oncodrive-FM) identified significantly mutated genes (Fig. 1a and Supplementary Data 4). *TP53* was the most frequently mutated gene in this cohort. A total of 79% (44/56) of the patients harbored 48 mutations in *TP53*, and 19% of the mutations were truncation mutations leading to the inactivation of *TP53*.

A total of 57% of the patients harbored mutations in genes of the receptor tyrosine kinase (RTK)/RAS pathway: *EGFR* (16%), *KRAS* (14%), *MET* (13%), *BRAF* (7%), *NF1* (5%), and *NRAS* (4%); the mutations of the genes were mutually exclusive (*P* = 0.00065, Monte Carlo simulation). *EGFR* was the most frequently mutated oncogene in the RTK/RAS pathway, and eight out of nine *EGFR* mutations were common and targetable mutations, including exon 19 deletions and exon 21 L858R. The rare mutation of *EGFR* was L861Q, located in the tyrosine kinase domain. 14% of the patients, less than conventional lung adenocarcinoma (LUAD), harbored *KRAS* mutations, which mostly involved codon 12. We identified seven *MET* mutations, and six of them could lead to exon 14 skipping, which was validated by RNA-seq. The *MET* mutation rate in spindle cell carcinoma was significantly higher than that in pleomorphic carcinoma (*P* = 0.012, Fisher’s exact test). Four patients harbored five *BRAF* mutations, with one N581S mutation and two V600E mutations. Three mutations identified in *NF1*, a suppressor of RAS, were all loss-of-function alterations (frameshift or stop gain variants), thus activating the RTK/RAS pathway.

A total of 27% of the patients harbored one or more mutations in genes of the phosphatidylinositol 3-kinase (PI3K) pathway: *PIK3CA* (13%), *PTEN* (9%), *AKT3* (5%), and *AKT1* (2%). Two hotspot mutations of *PIK3CA*, E542K in the helical domain and H1047R in the kinase domain, were each detected in two patients. Five of the six *PTEN* mutations were truncation mutations, resulting in the loss of function of *PTEN* and activating the PI3K pathway. In addition, we identified one oncogenic mutation in *AKT1*: E17K, a candidate therapeutic target for PSC.

Analyses of somatic copy number variations (SCNVs) revealed significant amplifications of 11q13.3 (containing *CCND1*), 8q24.21 (containing *MYC*), and 7q31.2 (containing *MET*) and significant deletions of 9p21.3 (containing *CDKN2A*) and 13q13.1 (containing *RB1*) (Fig. 1a, Supplementary Fig. 3, and Supplementary Data 5). *MET* amplifications and/or mutations were found in 20% of patients. Similar to other types of lung cancer, amplifications or deletions of genes in the cell-cycle pathway (*CCND1*, *CDKN2A*, and *RB1*) were frequently identified in PSC.

We performed mutational signature analysis on 56 PSC samples using the nonnegative matrix factorization (NMF) algorithm and identified three independent mutational signatures (A, B, and C), corresponding to reported mutational signatures 4, 13, and 6, respectively (Supplementary Fig. 4). In addition, the contributions of 30 known signatures to each sample are demonstrated in Fig. 1b. Signature 4 was prominent in a large proportion of samples, suggesting that tobacco exposure was in the etiology of PSC. Smokers were more likely to have signature 4 contributions (*P* = 6.013 × 10^−5^, Fisher’s exact test), and there was a significant correlation between signature 4 contributions and the TMB among smokers (*ρ* = 0.641, *P* = 1.082 × 10^−5^, Spearman’s correlation). Other important mutational signatures contributing to PSC included signatures 2 and 13, attributed to APOBEC enzymes, and signatures 3, 6, and 15, related to abnormalities in DNA maintenance.

### Common origin of the epithelial and sarcomatoid components

**Fig. 2:**
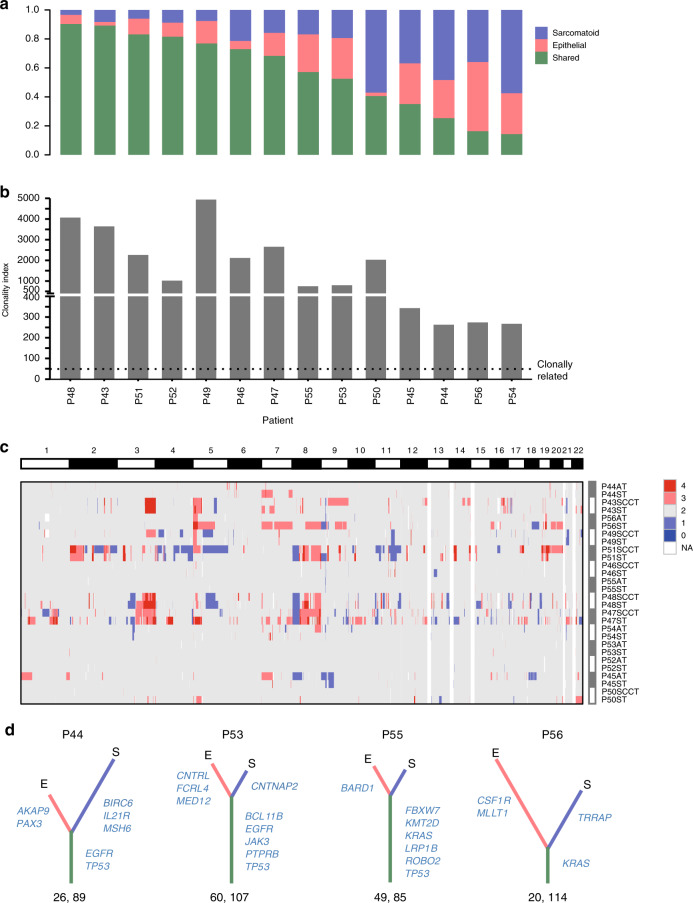
Establishing the clonal relationship between the epithelial and sarcomatoid components within the same tumor. **a** Columns depict the proportion of shared and specific somatic mutations of the 14 tumors microdissected. **b** Clonality indices for the 14 cases of PSC, suggesting the likelihood of common origin of the two components. Black dotted lines represent the cut-off value of clonality index to define clonal relatedness. **c** The distribution of copy number variations (CNVs) for all PSC samples microdissected. Red indicates CNV gain, and blue CNV loss. White represents the failure in CNV calling. AT, adenocarcinoma component; SCCT, squamous cell carcinoma component; ST, sarcomatoid component. Source data are provided as a Source data file. **d** Phylogenetic trees generated for 4 PSC samples. The length of the trunk (green) and branch (red or blue) represents the number of shared and specific nonsynonymous mutations, respectively. Part of driver mutations is marked. The number of truncal and total nonsynonymous mutations is indicated below. E and S represent epithelial and sarcomatoid component, respectively.

To explore the relationship between the epithelial and sarcomatoid components in the same PSC tumor, we analyzed somatic mutations and CNVs for both components. Somatic mutations were classified into two subgroups, shared (present in both components) and specific (present in only one component) mutations. We performed targeted sequencing and validated that the accuracy of specific mutation identification by WES was high. We also found that the specific mutations were enriched in non-loss of heterozygosity (LOH) regions, suggesting that LOH was not a confounding factor for specific mutation identification in this study. As demonstrated in Fig. 2a and Supplementary Table 1, the percentage of shared mutations ranged from 14 to 90% (median, 63%) in patients. Then, we calculated the clonality index (CI) of each tumor to assess the likelihood of a common origin of the two components and found that the epithelial and sarcomatoid components from each patient were clonally related (Fig. 2b and Supplementary Table 1)^13^. Furthermore, we calculated the percentage of CNV length shared by the two components to the aggregated CNV length of their union for each patient. The percentage of shared CNV length in ten patients was higher than 10% (range, 11.3–97.4%; median, 35.9%). Moreover, the distributions of somatic CNVs in the two components within the same tumor were similar (Fig. 2c). As a result, we inferred that the epithelial and sarcomatoid components of PSC originated from a common progenitor, and the transformation from epithelial to sarcomatoid components occurred during the carcinogenesis of PSC.

To illustrate the clonal architecture of PSC, we constructed phylogenetic trees using somatic mutations (Fig. 2d and Supplementary Fig. 5). The length of the trunk and branch represented the number of shared and specific mutations, respectively. Different trunk lengths in the phylogenetic trees implied the monoclonal origin of PSC. Mutations of *TP53*, *KRAS*, and *EGFR* were all in the trunk, suggesting they were early events in the carcinogenesis of PSC.

### EMT program in the carcinogenesis of PSC

Unsupervised hierarchical clustering of the transcriptome data from the epithelial and sarcomatoid components revealed that in most cases, two components from the same patient were in the same cluster (Supplementary Fig. 6), indicating that there were no substantial transcriptional changes in the transformation from epithelial to sarcomatoid components during carcinogenesis. We found a total of 195 differentially expressed genes (DEGs) between the epithelial and sarcomatoid components, and 36 of them were in a previously established 76-gene EMT signature^14^. DEGs between the two components were enriched in Kyoto Encyclopedia of Genes and Genomes (KEGG) pathways: ECM-receptor interaction, tight junction, focal adhesion, etc.; and Gene Ontology (GO) terms: cell–cell junction, extracellular matrix organization, cell adhesion, etc.; most of which were associated with epithelial and mesenchymal characteristics and EMT (Supplementary Fig. 7A).

**Fig. 3:**
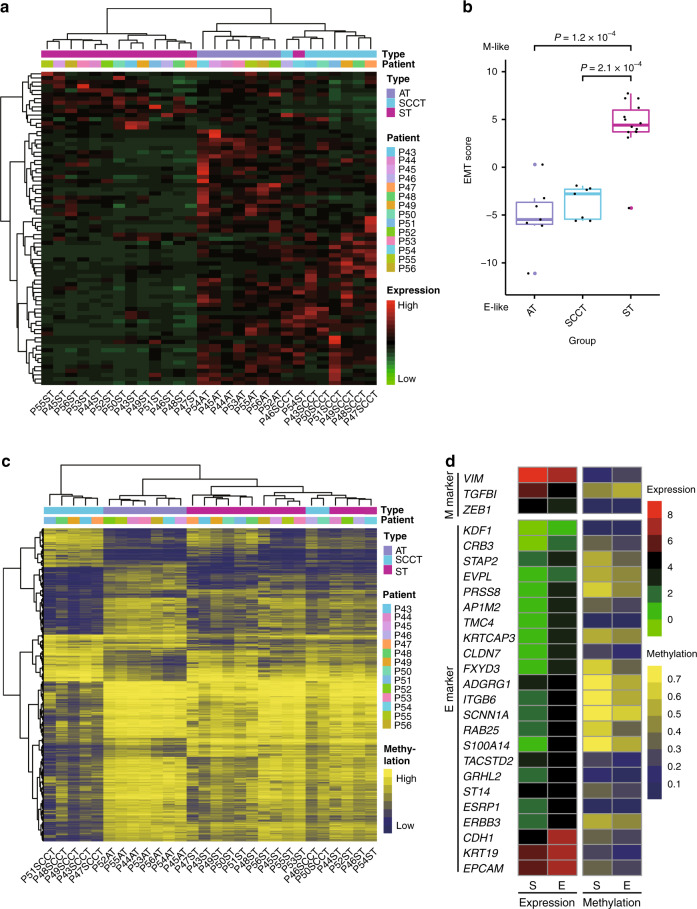
Transcriptional and DNA methylation profiles of the epithelial and sarcomatoid components. **a** The heatmap of hierarchical clustering with the expression level of a 76-gene EMT signature, annotated for the histological type and the patients. AT, adenocarcinoma component; SCCT, squamous cell carcinoma component; ST, sarcomatoid component. Source data are provided as a Source data file. **b** EMT scores are plotted for adenocarcinoma (AT), squamous cell carcinoma (SCCT), and sarcomatoid (ST) components as boxplots. Center line, median; box limits, upper and lower quartiles; whiskers, 1.5× interquartile range; colored points, outliers. Two-sided Wilcoxon rank-sum test was used for statistical analysis. No *P*-value adjustment was applied. *n* = 7, 7, and 14 for AT, SCCT, and ST group, respectively. Source data are provided as a Source data file. **c** The heatmap of hierarchical clustering with DNA methylation data of the epithelial and sarcomatoid components, annotated for the histological type and the patients. AT, adenocarcinoma component; SCCT, squamous cell carcinoma component; ST, sarcomatoid component. **d** The mean expression levels and DNA methylation levels of the 26 genes in the epithelial and sarcomatoid components. M (mesenchymal) markers and E (epithelial) markers are displayed in the upper and lower panel, respectively. S and E in the bottom represent sarcomatoid and epithelial components. Source data are provided as a Source data file.

EMT is an important phenomenon observed in several types of cancers and is associated with tumor aggressiveness and metastasis. Therefore, we explored the EMT status of the epithelial and sarcomatoid components based on transcriptome data. First, we performed clustering with the expression levels of the 76-gene EMT signature^14^, and two major clusters were yielded and dominant by sarcomatoid and epithelial components, respectively, suggesting distinct EMT statuses of the two components (Fig. 3a). Then, we calculated the EMT scores of each component from the 14 tumors to quantify the differences (Fig. 3b). The EMT scores of the sarcomatoid components were significantly higher than those of the ADC or SCC components (*P* = 1.2 × 10^−4^ and *P* = 2.1 × 10^−4^, respectively, Wilcoxon rank-sum test).

Unsupervised consensus clustering of DNA methylation data yielded three clusters, dominant by SCC, ADC, and sarcomatoid components, respectively, suggesting distinct DNA methylation profiles of sarcomatoid components (Fig. 3c). Genes containing differentially methylated probes (DMPs) between epithelial and sarcomatoid components were enriched in KEGG pathways: adherens junction, PI3K-Akt signaling pathway, focal adhesion, etc.; and GO terms: adherens junction, cell junction, regulation of cell–matrix adhesion, etc.; many of which correlated with EMT (Supplementary Fig. 7A). Therefore, we inferred that DNA methylation participated in the regulation of EMT, thus promoting tumor proliferation and metastasis. A total of 26 genes in the 76-gene EMT signature met the following criteria: (1) the expression levels were significantly up- or downregulated in the sarcomatoid components; (2) the methylation levels of more than 25% of the probes in the promoters were correspondingly down- or upregulated in the sarcomatoid components (Fig. 3d). For example, the expression level of *CDH1*, an epithelial phenotype marker, was downregulated (*P* = 4.8 × 10^−6^, Wilcoxon rank-sum test), while the methylation levels of *CDH1* promoter region were upregulated (*P* = 0.0001, Wilcoxon rank-sum test) in the sarcomatoid components, and the reverse situation was observed for *VIM*, a mesenchymal phenotype marker (Supplementary Fig. 7B-C).

### Tumor microenvironment of PSC

**Fig. 4:**
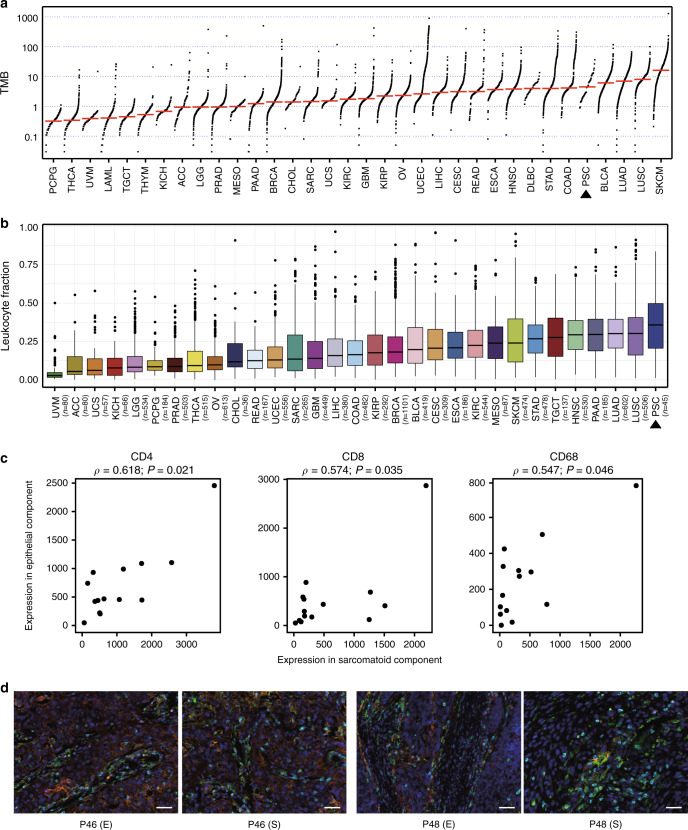
Tumor immune microenvironment of PSC. **a** Tumor mutation burden (TMB) of pulmonary sarcomatoid carcinoma (PSC) and The Cancer Genome Atlas (TCGA) tumor types, ordered by median. Somatic single-nucleotide variants and small indels of PSC were called using MuTect (version 3.1-0-g72492bb) and Strelka (version 1.0.14) via an in-house computational pipeline, and the mutation list of TCGA cancer types was released by the Pan-Cancer Atlas consortium (https://gdc.cancer.gov/about-data/publications/pancanatlas, mc3.v0.2.8.PUBLIC.maf.gz). **b** Leukocyte fraction (LF) of PSC and TCGA tumor types, ordered by median. The LF of all TCGA samples was collected from a previous study, and we estimated the LF of PSC using the algorithm provided by the same study^58^. Center line, median; box limits, upper and lower quartiles; whiskers, 1.5× interquartile range; points, outliers. **c** Spearman correlation of the fluorescence intensity of CD4, CD8, and CD68 between epithelial and sarcomatoid components. Source data are provided as a Source data file. **d** Representative images of fluorescent multiplex immunohistochemical analysis of P46 and P48. E and S represent epithelial and sarcomatoid components, respectively. Scale bar: 50 μm. The fluorescent multiplex immunohistochemical analysis was performed on the epithelial and sarcomatoid components of 14 patients. CD4, green; CD8, cyan; CD68, red; PD-L1, orange; FOXP3, magenta. CD4, cluster of differentiation 4; CD8, cluster of differentiation 8; CD68, cluster of differentiation 68.

High TMB, related to tumor neoepitope burden, and high LF, indicating a T-cell-inflamed TME, both act as predictive biomarkers for immunotherapy. Therefore, we performed a pan-cancer analysis of TMB and LF on 34 and 31 types of malignancies, respectively, to predict the efficacy of immunotherapy for PSC. As demonstrated in Fig. 4a and Supplementary Data 6, tumors within the top five TMB included skin cutaneous melanoma (SKCM), LUAD, lung squamous cell carcinoma (LUSC), bladder urothelial carcinoma (BLCA), and PSC, the majority of which are cancer types most responsive to immune checkpoint blockade. The TMB of PSC was 6.9 per megabase on average and lower than that of LUAD and LUSC (*P* = 0.037 and *P* = 2.545 × 10^−5^, respectively), and much higher than that of sarcoma (SARC) (*P* = 3.781 × 10^−13^), emphasizing the true epithelial nature. Tumors within the top three LF were PSC, LUSC, and LUAD (Fig. 4b and Supplementary Data 6), suggesting a T-cell inflamed microenvironment of PSC. The high TMB and LF indicated that immunotherapy would be a good treatment option for patients with PSC.

We performed fluorescent multiplex immunohistochemical analysis of PD-L1, CD4, CD8, CD68, and FoxP3 to quantitatively explore the relationship of the immune microenvironment between the epithelial and sarcomatoid components. There were no significant differences in the expression of the five markers between epithelial and sarcomatoid components. Significant correlations were observed in CD4, CD8, and CD68 expression between the two components (*ρ* = 0.618, *P* = 0.021; *ρ* = 0.574, *P* = 0.035; and *ρ* = 0.547, *P* = 0.046, respectively, Spearman’s correlation) (Fig. 4c). Representative images are shown in Fig. 4d and Supplementary Fig. 8. In consideration of the high LF of PSC, the epithelial and sarcomatoid components could both have rich immune cell infiltration. Although sarcomatous elements are generally considered to be resistant to treatment, however, they might not weaken the efficacy of immunotherapy. These results further highlighted the potential of immunotherapy for patients with PSC.

### Molecular classification

**Fig. 5:**
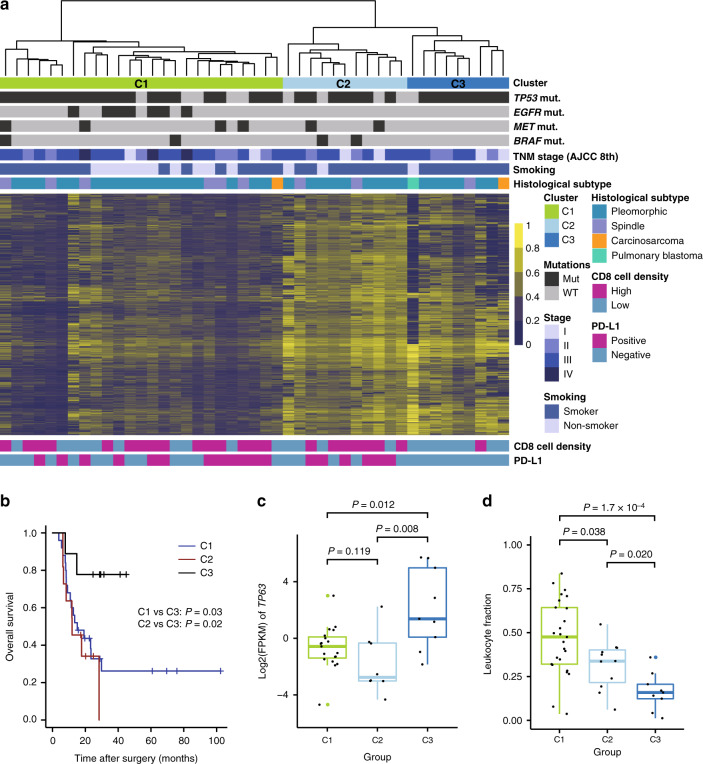
Integrated molecular classification of PSC. **a** PSC tumors are classified into subgroups based on DNA methylation patterns, annotated for the mutation status (Mut, mutated; WT, wild-type) of important genes, TNM stage, smoking status, and histological subtype in the top panel and for the immunohistochemical analysis of PD-L1 and CD8 in the bottom panel. **b** Kaplan–Meier analysis of C1, C2, and C3. The significance is determined by log-rank test. **c** Boxplot shows the expression level of *TP63* for three clusters. **d** Leukocyte fraction of each sample for three clusters are plotted as boxplots. Center line, median; box limits, upper and lower quartiles; whiskers, 1.5× interquartile range; colored points, outliers. Two-sided Wilcoxon rank-sum test was used for statistical analysis of (**c**) and (**d**). No *P*-value adjustment was applied. PD-L1, programmed death ligand 1; CD8, cluster of differentiation 8; *TP63*, tumor protein 63.

Considering the high level of intertumoral heterogeneity, molecular classification could provide insights into the biology, prognosis and treatment of PSC and improve clinical practice. Unsupervised hierarchical clustering of DNA methylation data yielded three major clusters: C1, C2, and C3 (Fig. 5a and Supplementary Data 7). A similar grouping was observed in the clustering analysis of transcriptome and DNA methylation data using the cross-platform iCluster tool (Supplementary Fig. 9). C1 had a relatively lower DNA methylation level than C2 and C3. We calculated Pearson correlations of the DNA methylation profiles between samples clustered above and microdissected samples and found that tumors in C1 had high correlations with ADC and corresponding sarcomatoid components (Supplementary Fig. 10), indicating the relevance in carcinogenesis. Most nonsmokers were in C1, and *EGFR* mutations were significantly enriched in C1 (*P* = 0.012, Fisher’s exact test). Moreover, we identified mutations of *MET* exon 14 skipping and *BRAF* V600E in patients in C1. In total, more than 50% of the patients in this group harbored actionable alterations, suggesting targeted therapy could be a good treatment option for patients in C1.

The EMT scores of C3 tended to be lower than those of C1 and C2 (Supplementary Fig. 11A), and C3 had the lowest *VIM* expression and the highest *CDH1* expression levels (Supplementary Fig. 11B-C), suggesting that C3 maintained epithelial characteristics to some extent. Univariate and multivariate analyses showed that patients in C3 had a significantly longer overall survival (OS) than those in C1 and C2 (Fig. 5b and Supplementary Table 2), which might be partly due to the lowest level of mesenchymal features. Moreover, the expression level of *TP63*, a biomarker for SCC, in C3 was significantly higher than that in the other clusters (Fig. 5c). Most patients in C3 were smokers and did not harbor mutations common in ADC, such as *EGFR* and *KRAS*, suggesting the relevance in carcinogenesis with SCC.

C2 had a relatively high DNA methylation level, similar to C3. However, the expression level of *TP63* of C2 was significantly lower than that of C3. In addition, we identified distinct mutation pattern between C2 and C3, as C2 had targetable *MET* exon 14 skipping mutations and *BRAF* mutations.

As immunotherapy has become an important part of the treatment of NSCLC, we tried to explore the differences in the immune microenvironment among the three clusters. C1 had a significantly higher expression level of *CTLA4* (*P* = 0.028, Wilcoxon rank-sum test) and *PD-L2* (*P* = 0.002, Wilcoxon rank-sum test) than C3 (Supplementary Fig. 11D-E). Then, we estimated the lymphocyte infiltration of each tumor by calculating the LF based on DNA methylation and LIexpression score based on the expression levels of a predefined 18-gene signature. As demonstrated in Fig. 5d and Supplementary Fig. 11F, C1 had the most infiltrating lymphocytes, while C3 had the least infiltrating lymphocytes. Immunohistochemical analysis demonstrated that 40% of the samples were positive for PD-L1 and that patients in C3 had lower expression of PD-L1 (*P* = 0.007, Fisher’s exact test) and lower cell density of CD8-positive lymphocyte infiltration (*P* = 0.010, Fisher’s exact test) than the other two clusters (Supplementary Fig. 12). The differences in the immune microenvironment among the three clusters would provide clues for the future clinical research on the refined immunotherapy for PSC.

### Integrated analysis of PSC, LUAD and LUSC

**Fig. 6:**
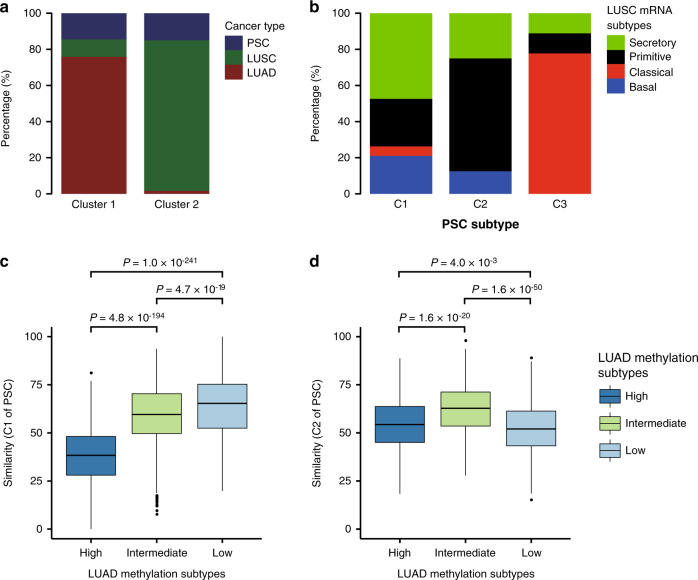
Integrated analysis of PSC, LUSC and LUAD. **a** The percentage of PSC, LUSC, or LUAD in the two major clusters yielded by unsupervised hierarchical clustering of DNA methylation data. **b** The proportion of the four LUSC subtypes in three clusters of PSC. Source data are provided as a Source data file. **c** Boxplots show the Euclidean distance-based similarity between the DNA methylation profiles of C1 and three LUAD methylation subtypes. Source data are provided as a Source data file. **d** Boxplots show the Euclidean distance-based similarity between the DNA methylation profiles of C2 and three LUAD methylation subtypes. Center line, median; box limits, upper and lower quartiles; whiskers, 1.5× interquartile range; points, outliers. Two-sided Wilcoxon rank-sum test was used for statistical analysis of (**c**) and (**d**). No *P*-value adjustment was applied. Source data are provided as a Source data file.

Unsupervised hierarchical clustering of DNA methylation data of PSC (detected by us) and LUAD and LUSC (released by TCGA) yielded two major clusters, dominant by LUSC and LUAD, respectively (Fig. 6a and Supplementary Fig. 13). PSC samples could be classified into two subgroups: PSC_SC and PSC_AD based on which cluster, LUSC or LUAD dominant, they were grouped into.

Most samples of the C3 subtype of PSC were in the PSC_SC subgroup, suggesting the relevance in carcinogenesis of C3 to LUSC. Further, to explore the relationship between the expression profiles of PSC and LUSC transcriptional subtypes, we calculated Pearson correlation coefficients between the predictor centroids for LUSC transcriptional subtypes and PSC samples, and the subtype prediction for each PSC sample was provided by the centroid with the maximum correlation coefficient^15^. In Fig. 6b, we demonstrated the proportion of the four LUSC subtypes in three clusters of PSC, and found that most of the samples in C3 were defined as the classical subtype. Moreover, we used the correlation coefficients between TCGA LUSC samples and the predictor centroids for each subtype as positive control (Supplementary Fig. 14), and PSC samples defined as the classical subtype were no less correlated with the corresponding centroid than LUSC samples, indicating that the expression profiles of C3 were similar to those of the classical subtype of LUSC. The above results provided clues for the biological similarities between C3 and the classical subtype of LUSC.

We performed unsupervised hierarchical clustering of DNA methylation data of PSC_AD and TCGA LUAD, and PSC_AD samples were divided into two subsets, dominant by samples in C1 and C2, respectively (Supplementary Fig. 15). Moreover, we evaluated the similarity between the DNA methylation profiles of C1 or C2 and three LUAD methylation subtypes by calculating the Euclidean distance (Fig. 6c-d), and found that PSC samples in C1 and C2 were more similar in DNA methylation profiles to CpG island methylator phenotype-low (CIMP-low) and CIMP-intermediate LUAD, respectively, suggesting the relevance in carcinogenesis^16^. In addition, an analysis similar to the LUSC transcriptional subtype prediction was performed to explore the relationship between the expression profiles of PSC and LUAD transcriptional subtypes (Supplementary Fig. 16). However, the correlation coefficients between PSC samples and three LUAD subtype predictor centroids were significantly lower than those between LUAD samples and the centroids, indicating distinct expression profiles between PSC and LUAD transcriptional subtypes.

## Discussion

This multi-omics analysis revealed the molecular characteristics, depicted the carcinogenesis and established the classification, providing clues for potential therapeutic strategies of PSC.

The somatic mutations of PSC were enriched in the p53, RTK/RAS, and PI3K pathways, and somatic CNVs were commonly observed in the cell-cycle pathway, which was reminiscent of the genomic alterations reported in conventional NSCLC^15,16^. *TP53* mutations were the most common mutation in our cohort, found in 79% of the patients, which was slightly higher than the frequency of 74% reported by Schrock et al. and Mehrad et al.^17,18^. Moreover, the TMB of PSC was within the top five in the pan-cancer analysis. The average TMB of our PSC cohort was 6.9 per megabase, lower than the 13.6 per megabase previously reported by Schrock et al., and the discrepancy could result from different variant calling algorithms and TMB calculation methods^18^. The molecular characteristics emphasized the true epithelial nature of PSC. Therefore, we inferred that the cell origin of PSC is similar to that of conventional NSCLC, which has been reported to originate from alveolar epithelial type 2 (AT2) cells, Clara cells, bronchioalveolar stem cells (BASCs), or basal cells^19^; however, further verification is required. In addition, the above results suggested that the recommended traditional treatment for conventional NSCLC, platinum-based chemotherapy, could be theoretically feasible for PSC.

However, PSC exhibited a high rate of resistance to traditional chemotherapy^3^. Therefore, seeking new therapeutic targets is an important issue to improve patient outcomes. A total of 16% of the patients harbored *EGFR* mutations, and 89% of them were common mutations and sensitive to EGFR tyrosine kinase inhibitors (TKIs). The frequency of common *EGFR* mutations was higher than that reported in studies on the Western population^17,18,20^, and was similar to that reported for an Asian cohort^21^, suggesting the ethnic differences. We identified one rare mutation of *EGFR*, L861Q, which was less sensitive to EGFR-TKIs^22^. However, patients with an L861Q mutation might benefit from the second generation targeted agent afatinib^23^. We observed a higher mutation rate of *MET* in PSC than in conventional LUAD (13% versus 6%)^16^. *MET* exon 14 skipping mutations were previously reported in 0% to 22% of PSC cases, and the highly variable frequencies could be due to the different methodologies and limited sample sizes^9,17,18,20,21^. We identified *MET* exon 14 skipping mutations and *MET* DNA alterations in 11% and 20% of our cohort, respectively, making these patients potential beneficiaries of crizotinib^9^. In addition, we found that 7% of the patients harbored *BRAF* mutations, including two V600E mutations, which were sensitive to the combined therapy of dabrafenib and trametinib^24^. In total, 27% of the patients had mutations in the PI3K pathway, suggesting the abnormal activation of downstream elements. Multiple PI3K pathway inhibitors are under clinical research and could be a treatment option for these patients^25–27^. One of the mutations in the PI3K pathway was the E17K mutation of *AKT1*, which is an oncogenic mutation and could be a therapeutic target for AZD5363, a pan-AKT kinase inhibitor^28^. Our study demonstrated that a large proportion of patients with PSC could benefit from targeted therapy; however, prospective randomized controlled trials are needed to provide more evidence.

The genomic alterations, transcriptional profiles and DNA methylation profiles of epithelial and sarcomatoid components revealed high intratumoral heterogeneity at the molecular level. However, a large number of shared mutations and a considerable length of shared CNVs were identified in the two components, and analysis of clonality indices revealed that the two components from each patient were clonally related. Therefore, we inferred the monoclonal origin of PSC. Although the conclusion was consistent with that of previous studies^20,29,30^, which demonstrated that the epithelial and sarcomatoid components shared the same *TP53*, *KRAS*, and *EGFR* mutations, we provided direct evidence based on global genomic profiles with the largest sample size. We also showed that the epithelial and sarcomatoid components of the microdissection samples shared driver mutations (such as *KRAS* and *EGFR* mutations) that were commonly observed in conventional lung cancer but were rare in sarcoma, further emphasizing the true epithelial nature of PSC and suggesting that the transformation of the epithelial components might occur first. *EGFR* mutations were all in the trunk of the phylogenetic trees, suggesting the epithelial and sarcomatoid components could both respond to EGFR-TKIs, leading to possible favorable outcomes for these patients regarding targeted therapy.

Exploring the molecular pathogenesis of PSC remains an important issue in this field. Based on immunohistochemistry (IHC) analysis of several EMT markers, Pelosi et al. and Blaukovitsch et al. proposed that the sarcomatoid component of PSC might undergo EMT^31,32^. In this study, we demonstrated the transcriptional and DNA methylation profiles of the epithelial and sarcomatoid components. The DEGs between the two components were enriched in pathways and terms related to EMT, with sarcomatoid components exhibiting a higher degree of mesenchymal characteristics, which could explain the high aggressiveness and drug resistance of PSC. As sarcomatoid components had distinct DNA methylation profiles, and the association between DNA methylation levels and expression levels was observed in 26 genes of the 76-gene EMT signature, epigenetic regulation might participate in the EMT process and the carcinogenesis of PSC. Several small molecule inhibitors and miRNAs have been reported to reverse the EMT phenotype in several types of cancer^33^. Therefore, EMT could be a potential therapeutic target for PSC in the future, especially in the setting of combined therapy, as inhibiting EMT could overcome drug resistance.

Recent advancements in immunotherapy have opened a new avenue for the treatment of cancers, including NSCLC. TMB and tumor infiltrating lymphocytes (TILs) were reported to be predictive biomarkers for immune checkpoint blockade therapies^34,35^. Pan-cancer analysis revealed that PSC was among the top list of TMB and LF, indicating high neoantigen burden and a T-cell-inflamed TME. Considering the similar TME of the epithelial and sarcomatoid components from the same patient demonstrated by fluorescent multiplex IHC, the two components could both have a possible favorable response to immunotherapy. In addition, cases of PSC with dramatic response to immune checkpoint inhibitors were reported^36,37^, despite the lack of large-scale clinical research. Therefore, immunotherapy could offer a new hope for patients with PSC and help improve patient outcomes in the future.

PSC is a general term for five subtypes that exhibit different histological morphologies, suggesting high intertumoral heterogeneity. As a result, molecular classification is essential to deepen our understanding of the biology, predict prognosis, and guide treatment for PSC. On the basis of our classification, C1 had a relatively low DNA methylation level, and the carcinogenesis of C1 might be relevant to ADC. *EGFR* mutations were significantly enriched in C1, and *MET* and *BRAF* mutations were also identified, suggesting targeted therapy would be a good treatment option for patients in C1. The methylation level of C2 was high, and analysis of the TME demonstrated a T-cell inflamed microenvironment in C1 and C2, providing clues for the future clinical research on the refined immunotherapy for PSC. The carcinogenesis of some samples in C3 might have relevance to SCC because of the higher expression level of *TP63*. Moreover, C3 maintained the highest level of epithelial status, and patients in this group had a significantly longer OS than patients in C1 and C2. Therefore, the molecular classification could differentiate prognosis and provide evidence for future refined stratification in the treatment of PSC.

Integrated analysis of PSC, LUSC and LUAD demonstrated that PSC showed similarities in DNA methylation profiles to LUSC and LUAD, further emphasizing the true epithelial nature. The C3 subtype of PSC exhibited a DNA methylation pattern similar to that of LUSC and transcriptional profiles similar to those of the classical subtype of LUSC, while PSC samples in C1 and C2 were more similar in DNA methylation profiles to CIMP-low and CIMP-intermediate LUAD, respectively, suggesting the relevance in carcinogenesis. We inferred that these LUSC and LUAD subtypes might have greater potential to undergo EMT and sarcomatoid transformation; however, further investigation is required.

In conclusion, our study delineated the landscape of genomic alterations in PSC, emphasizing the epithelial nature and suggesting targeted therapy could be a good treatment option. In addition, the epithelial and sarcomatoid components within the same tumor shared a common origin and exhibited a distinct EMT status, which could be epigenetically regulated, suggesting the reversal of EMT as a potential therapeutic entry point. The pan-cancer analysis demonstrated the high TMB and LF of PSC, indicating applicability of immunotherapy. Furthermore, the molecular classification could deepen our understanding of the biology of PSC, predict prognosis and guide stratified treatment in clinical practice. Finally, the integrated analysis of PSC, LUSC and LUAD provided clues to their relevance in carcinogenesis.

## Methods

### Study design

We enrolled 56 patients diagnosed with PSC who underwent surgery at Cancer Hospital, Chinese Academy of Medical Sciences. Patients were selected only if they had no prior treatment before surgery and if they had tumor and paired normal lung tissue samples available. The clinical data of the 56 patients were obtained from hospital records. The diagnoses were confirmed by two independent expert pathologists according to the 2015 World Health Organization’s classification^2^. WES, transcriptome sequencing and DNA methylation detection were performed to delineate the genomic alterations, evaluated the TME and establish the molecular classification of PSC. Fourteen of the tumor samples simultaneously had sarcomatoid and epithelial components and were chosen for microdissection using the LMD7 system (Leica). We assessed the clonal relatedness and compare the transcriptional profiles, DNA methylation profiles and tumor immune microenvironment of the two components by integrated molecular characterizations. The Ethics Committee of National Cancer Center/Cancer Hospital, Chinese Academy of Medical Sciences and Peking Union Medical College approved this study (18-224/1782). Informed consent was obtained from each participant. The study was performed in accordance with the local ethical regulations and the guidelines of the Declaration of Helsinki.

### WES and analysis

We extracted total DNA from fresh frozen and formalin-fixed paraffin-embedded (FFPE) samples using the QIAamp DNA Mini Kit (Qiagen, cat. no. 51304) and QIAamp DNA FFPE Tissue Kit (Qiagen, cat. no. 56404), respectively. WES libraries were prepared using Agilent’s SureSelect Human All Exon V5 Kit (Agilent Technologies, cat. no. 5190-6209) according to the manufacturer’s instructions. DNA libraries were sequenced on the Illumina HiSeq or NovaSeq platform with a paired-end 2 × 150 protocol aiming for the coverage of 200× and 100× for tumor and normal DNA, respectively.

Raw data were trimmed and filtered using Trimmomatic 0.33^38^. Paired-end clean reads were aligned to the human reference sequence hg19 using the BWA-MEM algorithm (BWA version 0.7.10-r789) with default parameters^39^. Somatic single-nucleotide variants (SNVs) and small indels were called using MuTect (version 3.1-0-g72492bb) and Strelka (version 1.0.14)^40,41^. All mutations in coding regions were manually checked using the Integrative Genomics Viewer (version 2.3.34)^42^. sCNVs were called using Control-FREEC (version 10.5)^43^. Significant focal copy number alterations were identified using GISTIC 2.0^44^.

We used two methods to identify the mutational processes active in PSC. The deconstructSigs package (v1.8.0) was used to infer the contributions of 30 published signatures from the Catalog of Somatic Mutations in Cancer (COSMIC) (https://cancer.sanger.ac.uk/cosmic/signatures_v2) in each tumor^45^. We also used NMF to predict signatures and compared the identified signatures with the 30 known signatures in COSMIC^46^.

We used four algorithms to identify significantly mutated genes (SMGs): MutSigCV (version 1.41; Benjamini–Hochberg false discovery rate (FDR) *q*-value <0.1), the SMG test from MuSiC (v0.4; at least two FDR ≤ 0.2), OncodriveCLUST (V0.4.1; *q*-value <0.05), and Oncodrive-FM (version 1.0.3; *q*-value <0.05)^47–50^.

The TMB was calculated as the number of somatic SNVs and indels per megabase of the coding region. We calculated the TMB for all The Cancer Genome Atlas (TCGA) samples based on the mutation data released by the Pan-Cancer Atlas consortium, available on their public page (https://gdc.cancer.gov/about-data/publications/pancanatlas, mc3.v0.2.8.PUBLIC.maf.gz).

We performed targeted sequencing (average sequencing depth: 3229×) and checked whether a specific mutation was exclusively identified in only one component of the same tumor. By this procedure, 85% (40/47) of the covered specific mutations were confirmed as truly specific mutations. For three of the remaining specific mutations, the allele frequencies were much lower in the components, in which WES did not identify the specific mutations, but the tumor purity reviewed by pathologists was similar, indicating the true intratumoral heterogeneity. The other four mutations were excluded from further analysis, as (1) the depth of targeted sequencing was extremely low (1 mutation); (2) the private mutation resulted from random sampling of sequencing reads (1 mutation); or (3) the private mutations were not detected in targeted sequencing (2 mutations).

To assess whether the epithelial component and the matching sarcomatoid component of a given case were clonally related, we calculated the CI for each microdissection sample. CI was defined as ${\mathrm{CI}} = - {\mathrm{log}}_{10}\mathop {\prod }\limits_{m = 1}^M {\mathrm{P}}({\mathrm{X}})_m$. *M* represented the number of identical mutations in the two given samples, and P(X) represented the probability of a given mutation being observed in both samples. P(X) was defined as ${\mathrm{P}}\left( {\mathrm{X}} \right) = C_n^kp^k(1 - p)^{n - k}$, *n* = 2, *k* = 2, and the definition of *p* was as previously described^13^, except that the unrelated PSC cohort was used instead of the TCGA EECs cohort. To define a cut-off of CI for clonal relatedness, 40%, 60% and 80% of the mutations from the unrelated PSC samples were randomly selected in duplicate as the positive control, and an equivalent number of pairs of the unrelated PSC samples were randomly selected as the negative control. The R package “ROCR” (v1.0-7) was used to define the optimum cut-offs. We repeated the above procedures 100 times to obtain the median and the 95% confidence interval of the optimum cut-off.

### RNA-seq and analysis

RNA was extracted from fresh frozen samples using TRI Reagent (Sigma-Aldrich, cat. no. T9424) and the RNeasy MinElute Cleanup Kit (Qiagen, cat. no. 74204). High-quality RNA samples were processed with TruSeq Stranded Total RNA HT- (with Ribo-Zero TM Gold) (Illumina, cat. no. RS-122-2303) under the guidance of the TruSeq Stranded Total RNA Sample Preparation Guide. We extracted RNA from FFPE samples using the RNeasy FFPE Kit (Qiagen, cat. no. 73504), and the sequencing libraries of the RNA samples extracted from FFPE tissues were prepared using the TruSeq RNA Exome, composed of TruSeq RNA Library Prep for Enrichment (Illumina, cat. no. 20020189), TruSeq RNA Enrichment (Illumina, cat. no. 20020490), Exome Panel (Illumina, cat. no. 20020183), and TruSeq RNA UD Indexes (Illumina, cat. no. 20022371). RNA sequencing was performed using the Illumina HiSeq platform following protocols for 2 × 150 bp paired-end sequencing aiming for 14G and 6G raw data for RNA from fresh frozen and FFPE tissues, respectively.

Raw data were trimmed and filtered using Trimmomatic 0.33. We followed the HISAT, StringTie, and the R package Ballgown protocol for gene expression quantification^51–53^. Paired-end reads were aligned to the human reference sequence GRCh38 using HISAT2 (version 2.0.5). BAM files were sorted and indexed by SAMtools (version 1.3)^54^. Then, StringTie (version 1.3.1c) was used to assemble transcripts, estimate transcript abundances and create table counts for each sample. Finally, Ballgown (version 2.8.4) was used to extract gene-level expression measurements from the Ballgown objects obtained from StringTie. Cyber-T was used to call DEGs (fold-change ≥2, *P* value <0.05)^55^. Functional enrichment analysis was performed using KOBAS v3.0^56^.

For microdissected samples, we identified the 2000 most variably expressed genes using the median absolute deviation. Hierarchical clustering was performed with the 2000 genes using Euclidean distance for clustering and Ward’s method for linkage.

The first principal component of the expression data of a previously established 76-gene EMT signature was used as the EMT score, representing the EMT status of the samples^14^. The principal components analysis was performed with the R package FactoMineR (version 1.42). We obtained LIexpression scores by running single-sample gene set enrichment analysis (ssGSEA) with the expression levels of a predefined 18-gene signature representing overall lymphocyte infiltration^57,58^. The R package GSVA (version 1.30.0) was applied in the analysis.

The subtype predictor centroids for LUSC^59^ and LUAD^60^ were used to make transcriptional subtype predictions for PSC following a nearest centroid procedure. We calculated Pearson correlations between the predictor centroids and PSC samples using the genes common to the predictor and our PSC cohort (197 genes for LUSC, 473 genes for LUAD), and the transcriptional subtype prediction for each PSC sample was provided by the centroid with the maximum correlation coefficient.

### DNA methylation detection and data processing

DNA methylation profiles were examined using the Infinium MethylationEPIC Kit (Illumina, cat. no. WG-317-1001) or HumanMethylation450 BeadChip Kit (Illumina, cat. no. WG-314-1003) following the manufacturer’s protocol. The obtained raw IDAT files (two per sample) were directly processed by the R/Bioconductor package ChAMP (version 2.9.10) with default parameters, and *β* values were recorded^61,62^. A probe was filtered if: (i) a detection *P* value was above 0.01 in at least one sample; (ii) a beadcount <3 was found in at least 5% of samples; (iii) it was a non-cg probe; (iv) single-nucleotide polymorphisms (SNPs) were found in it; (v) it was aligned to multiple sites; and (vi) it was on the X or Y chromosome.

DMPs were assessed using the empirical Bayes method called limma (version 3.34.9). A probe was defined as a DMP if the adjusted *P* value was ≤0.05 and the difference between median *β* values was ≥0.3 (|Δ*β*| ≥0.3).

For molecular classification, we selected cancer-specific DNA hypermethylation probes, which were unmethylated in the normal samples (median *β* value <0.2) and methylated in the tumor samples (median *β* value >0.3). For the microdissected samples, we selected the union of the DMPs from four contrasts (paired sarcomatoid and ADC components, paired sarcomatoid and SCC components, unpaired sarcomatoid components between ADC-containing patients and SCC-containing patients and unpaired ADC and SCC components). Then, hierarchical clustering was performed using Euclidean distance for clustering and Ward’s method for linkage.

We performed unsupervised hierarchical clustering of DNA methylation data of PSC, LUSC and LUAD with the union of DMPs between PSC, LUSC, or LUAD tumor samples and their corresponding paired normal lung tissues, respectively. Among the probes, which were significantly differentially methylated between LUAD tumor samples and paired normal lung tissues (adjusted *P* value ≤0.05), we screened out probes meeting the criteria that the absolute value of the difference of average *β*-value between LUAD tumor samples and corresponding normal lung tissues was greater than or equal to 0.2 (|Δ*β*| ≥0.2), and on this basis, we further selected the 3000 most variably methylated probes using the median absolute deviation to perform hierarchical clustering of PSC_AD and TCGA-LUAD samples. The annotation for subtypes and the DNA methylation data of LUSC and LUAD were collected from previous TCGA studies^15,16^.

We estimated the LF of each sample using a mixture model^58^. We identified 2000 DNA methylation loci that were the most differentially methylated between normal lung tissues and leukocyte, 1000 in each direction. Assuming two populations for each locus *i*, for each sample we have $\beta _i = \beta _{i{\mathrm{L}}}\pi + \beta _{i{\mathrm{T}}}(1 - \pi )$. $\beta _i$ denoted the *β* value for probe *i* in tumor sample. $\beta _{i{\mathrm{L}}}$ and $\beta _{i{\mathrm{T}}}$ denoted the *β* value of leukocyte and pure tumor for the locus *i*, respectively. *π* denoted the fraction of leukocyte components. Tumor with the least evidence of leukocyte methylation was used as a surrogate for the *β* value for each locus in the pure tumor. The DNA methylation data of peripheral blood mononuclear cells (PBMCs) from six healthy donors (GSE35069) were used as the source of the leukocyte DNA methylation level^63^. With the measured *β* value for tumor and leukocyte, we solved for *π* for each locus. The LF was calculated as the mode of kernel density estimate. The LF of all TCGA samples was collected from a previous study^58^.

### Integrative clustering using iCluster

We used the cross-platform iCluster tool^64^ (R package iClusterPlus v1.16.0) to perform unsupervised clustering analysis of PSC on transcriptome and DNA methylation data. For the transcriptome data, we employed the median absolute deviation to select the top 2000 most variably expressed genes after log2 transformation and median centering by gene. For the DNA methylation data, we selected the top 2000 most variable CpG sites from the cancer-specific hypermethylation probes. The four-cluster result was chosen to present.

### IHC and fluorescent multiplex IHC

The tissue microarray (TMA) was manufactured with two cores of 2 mm taken from each sample and used for the IHC of CD8 and PD-L1. The TMA was incubated with primary antibodies against CD8 (D8A8Y, 1:1000, Cell Signaling Technology, cat. no. 85336) and PD-L1 (28-8, 1:500, Abcam, cat. no. ab205921), followed by incubation with the secondary antibodies and 3,3′-diaminobenzidine. PD-L1 tumor positivity was defined as a ≥ 1% membranous tumor proportion score (TPS). Tumors were divided into high- and low-infiltrated groups with the cut-off value as the median averaged count per high-power field for each case of CD8-positive lymphocytes.

We performed fluorescent multiplex IHC of PD-L1, CD4, CD8, CD68, and FoxP3 on the sections from the 14 samples microdissected. The procedures of primary antibody incubation, secondary antibody incubation and tyramide signal amplification (TSA) visualization were repeated for each antigen. Primary antibodies against PD-L1 (E1L3N, 1:100, Cell Signaling Technology, cat. no. 13684), CD4 (EP204, 1:100, Zsbio, cat. no. ZA-0519), CD68 (KP1, 1:500, Zsbio, cat. no. ZM-0060), and FoxP3 (236A/E7, 1:100, Abcam, cat. no. ab20034) were incubated at 37 °C for 1 h, and primary antibody against CD8 (SP16, 1:100, Zsbio, cat. no. ZA-0508) was incubated at 4 °C overnight. We performed TSA visualization with the Opal Seven-color IHC Kit (PerkinElmer, cat. no. 2665291) and TSA Coumarin system (PerkinElmer). Slides were scanned using the PerkinElmer Vectra. Representative original multispectral images were used to train the inForm software (version 2.3.0), and the settings were saved to allow batch analysis of multispectral images of the same tissue^65^.

### Validation of somatic mutations

The somatic mutations of *TP53*, *EGFR*, *KRAS*, *PIK3CA*, *MET*, *PTEN*, *BRAF*, and *NF1* were validated by PCR-Sanger sequencing and/or RNA-seq. RNA-seq reads with a mapping quality <20 and PCR duplication reads were removed using SAMtools^54^. Then, SAMtools mpileup and BCFtools (version 1.3.1, https://samtools.github.io/bcftools/) were combined to call SNVs and indels for each tumor and adjacent normal tissue (if available). Somatic mutations are validated if they are called in only tumor RNA-seq data.

### Statistics analysis

Statistical analysis was performed using Fisher’s exact test for categorical variables, and the Wilcoxon test or Mann–Whitney U test for continuous variables. We estimated the mutual exclusivity of mutations by Monte Carlo simulation. We calculated the relevance between continuous variables using Pearson’s correlation or Spearman’s correlation. Kaplan–Meier curves and the log-rank test were used for the survival analysis. Statistical tests were performed in R (version 3.2.0). We regarded a two-tailed *P* value <0.05 as statistically significant.

### Reporting summary

Further information on research design is available in the Nature Research Reporting Summary linked to this article.

## Supplementary information

Supplementary Information

Description of Additional Supplementary Files

Supplementary Data 1

Supplementary Data 2

Supplementary Data 3

Supplementary Data 4

Supplementary Data 5

Supplementary Data 6

Supplementary Data 7

Reporting Summary

## Data Availability

WES and RNA-seq data have been deposited to Genome Sequence Archive (GSA) in BIG Data Center, Beijing Institute of Genomics (BIG) under accession number HRA000298. The clinical data are provided in Supplementary Data 1. A complete list of somatic nonsynonymous mutations can be found in Supplementary Data 3. The data supporting Figs. 2a, 2b, 4a, 4b, 5a, 5c, and 5d and Supplementary Figs. 3, 7B, and 11 of the study are available in the Supplementary Data files. The DNA methylation data of PBMCs were obtained from the GEO dataset with accession number: GSE35069. The transcriptome and DNA methylation data of TCGA LUAD and LUSC were collected from the following web-links https://portal.gdc.cancer.gov/projects/TCGA-LUAD and https://portal.gdc.cancer.gov/projects/TCGA- LUSC, respectively. All the other relevant data of the study are available from the corresponding authors upon reasonable request. Source data are provided with this paper.

## Source data

Source Data
