# Supplementary material for: Integrated molecular characterization reveals potential therapeutic strategies for pulmonary sarcomatoid carcinoma
Source: Nat Commun. 2020 Sep 28;11:4878. doi: 10.1038/s41467-020-18702-3 (PMC7522294; doi:10.1038/s41467-020-18702-3)
Supplement: Supplementary file 1 — Supplementary Information [file 41467_2020_18702_MOESM1_ESM.pdf]

Supplementary Information

Yang et al.: Integrated molecular characterization reveals potential therapeutic strategies for pulmonary sarcomatoid carcinoma.

Supplementary Table 1. Somatic Mutations and Clonal Relatedness of the Epithelial and Sarcomatoid Components

Patient	Shared mutations	Specific mutations (Epithelial)	Specific mutations (Sarcomatoid)	Clonality index
P43	430	12	40	3641.479282
P44	31	32	59	262.7561908
P45	40	32	42	343.1461922
P46	208	16	61	2114.289073
P47	268	62	62	2659.17326
P48	421	29	16	4068.168041
P49	525	106	52	4936.928477
P50	206	12	289	2030.088881
P51	222	29	16	2262.394788
P52	93	11	10	1017.426763
P53	73	39	27	794.0949618
P54	25	49	100	267.4184856
P55	68	31	20	747.1335555
P56	25	73	55	273.8124221

Supplementary Table 2. Multivariate Analysis of Molecular Classification and TNM Stage

Variable		Multivariate Analysis	
		HR (95% confidence interval)	*P* Value
Molecular classification	C3	0.22 (0.05-0.95)	0.0428
	C1+C2		
TNM stage	I+II	0.20 (0.07-0.55)	0.0019
	III+IV		

The hazard ratios (HR) and *P* values were computed by COX proportional hazards model. No *P* value adjustment was applied. n = 9, 36 for subgroups C3 and C1+C2, respectively. n = 17, 28 for subgroups I+II stage and III+IV stage, respectively.

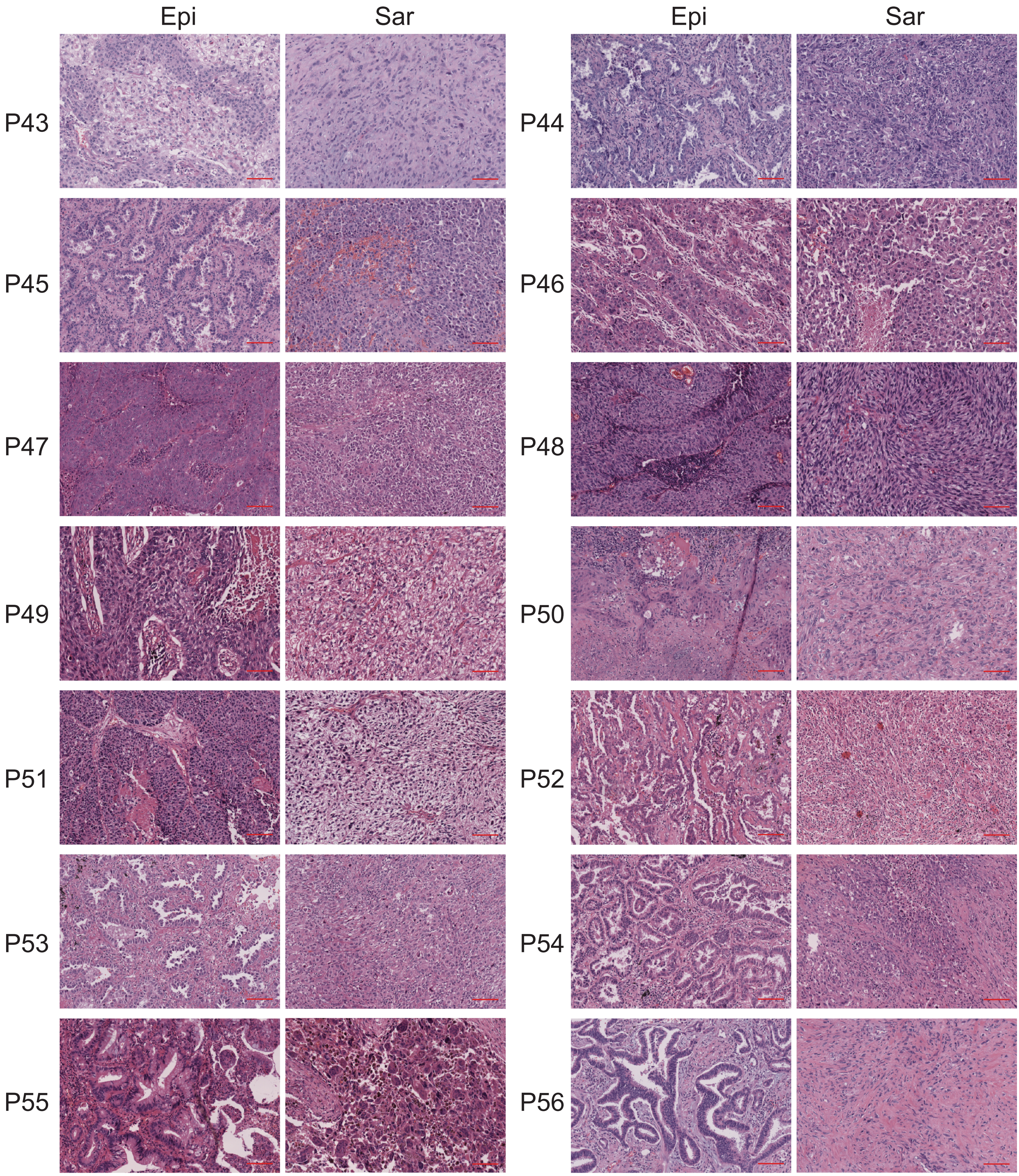

Supplementary Figure 1. Images of H&E staining for the epithelial and sarcomatoid components of all 14 microdissection samples. Scale bar: 100μm. Epi, epithelial component; Sar, sarcomatoid component.

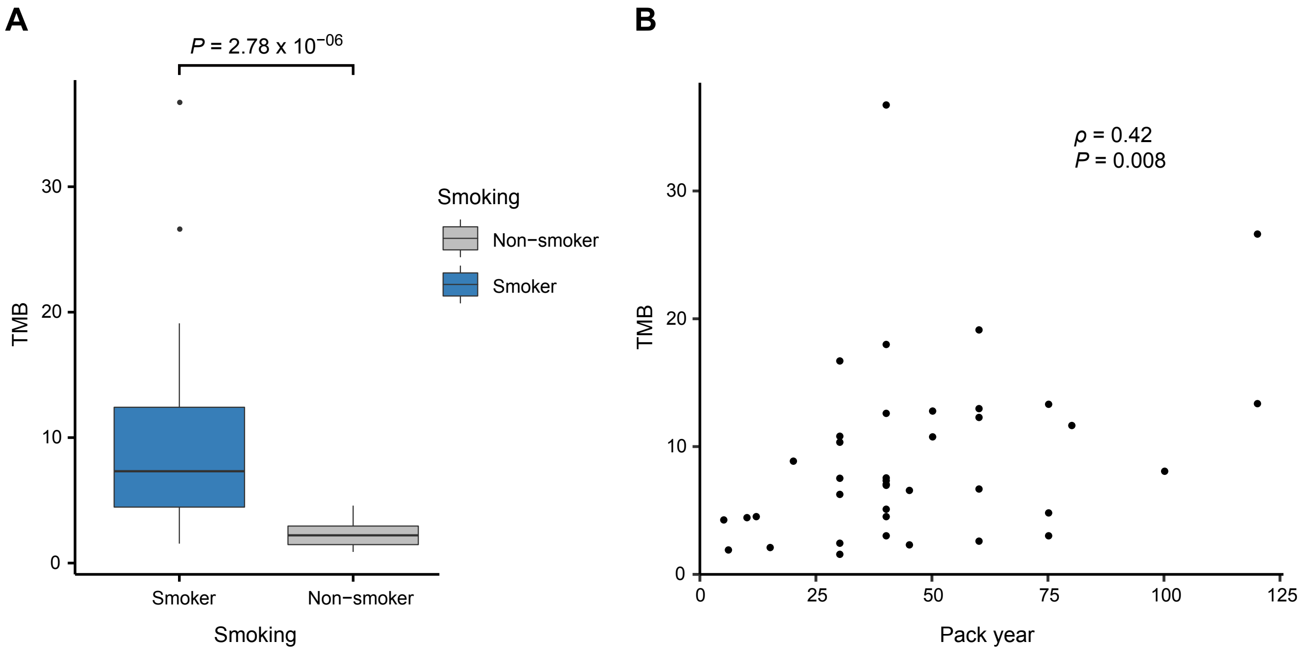

Supplementary Figure 2. Relationship between tumor mutation burden (TMB) and smoking. (A) Boxplot shows the TMB for smokers and nonsmokers. Center line, median; box limits, upper and lower quartiles; whiskers, 1.5× interquartile range; points, outliers. Two-sided Wilcoxon rank sum test was used for statistical analysis. No *P*-value adjustment was applied. $n = 39$, 17 for smokers and nonsmokers, respectively. (B) Spearman correlation between TMB and pack year of smoking among smokers.

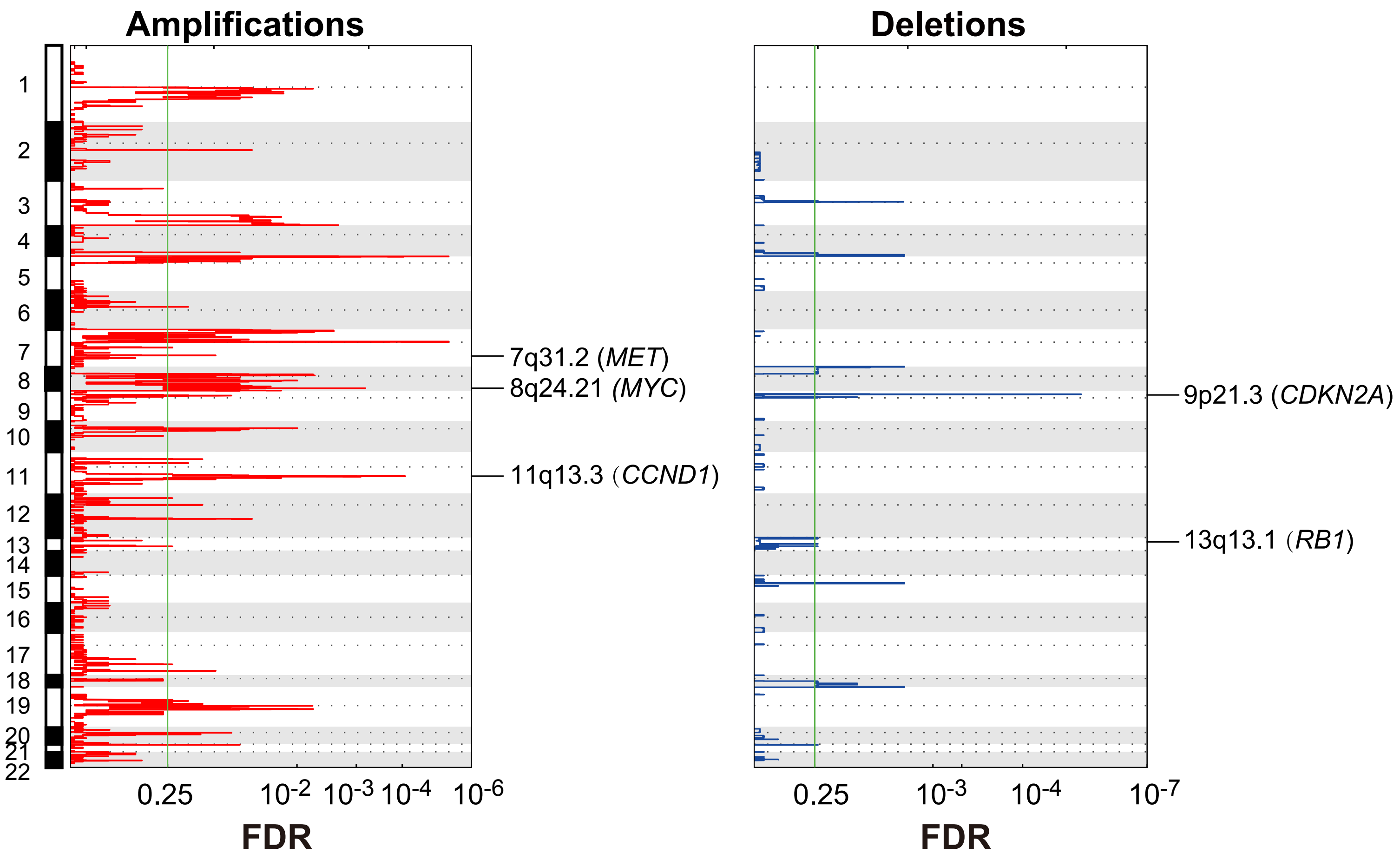

Supplementary Figure 3. Recurrent focal copy number variations in the 56 PSC samples by GISTIC analysis. The significance threshold (FDR 0.25) is indicated by green line for focally amplified and deleted regions. The *q* values were obtained directly from GISTIC.

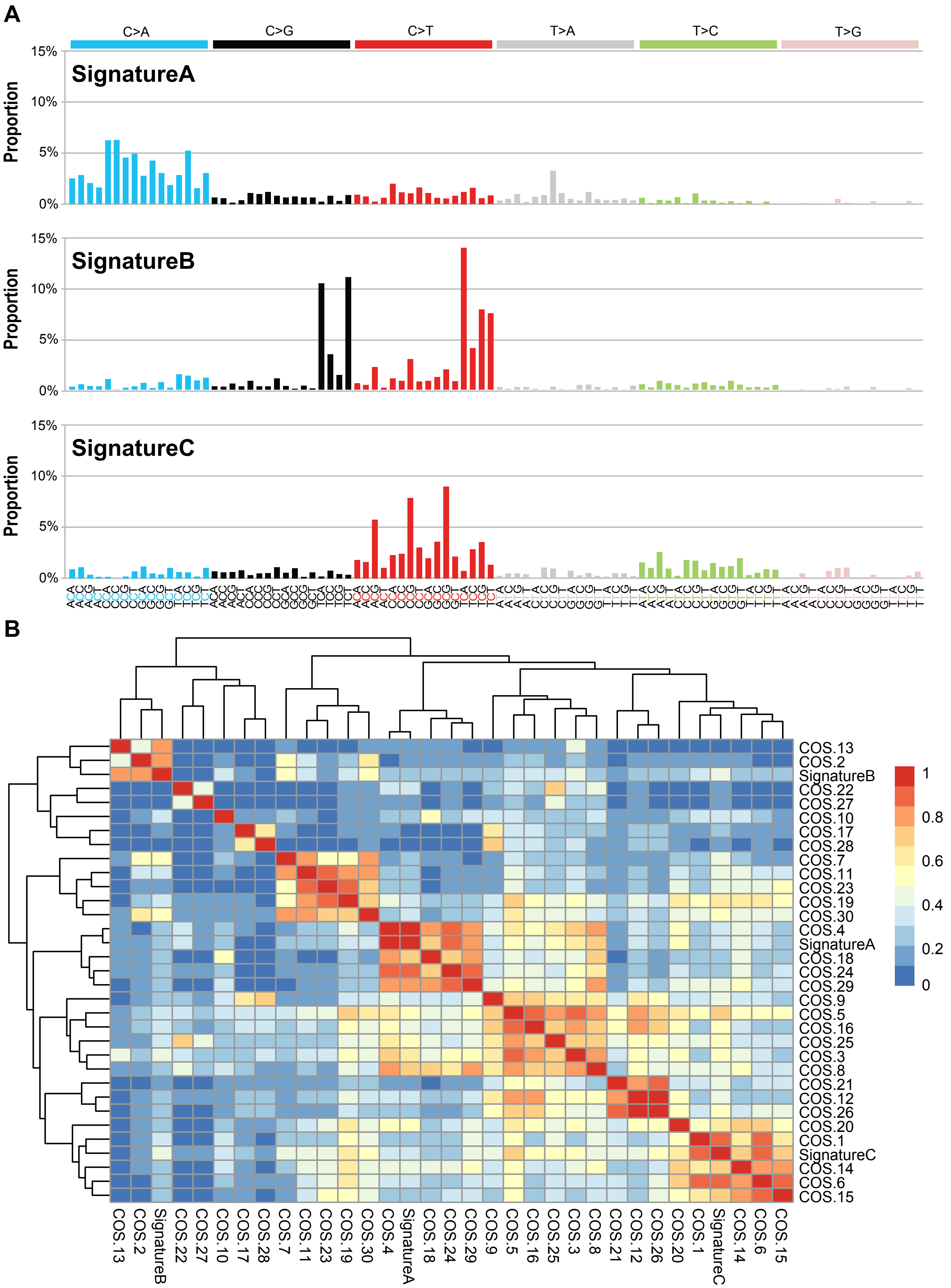

Supplementary Figure 4. Mutational signatures extracted from 56 PSC samples using nonnegative matrix factorization. (A) The charts show three mutational signatures of all tumor samples: signature A, signature B and signature C. (B) Cosine similarities heatmap of the three identified signatures and 30 known signatures.

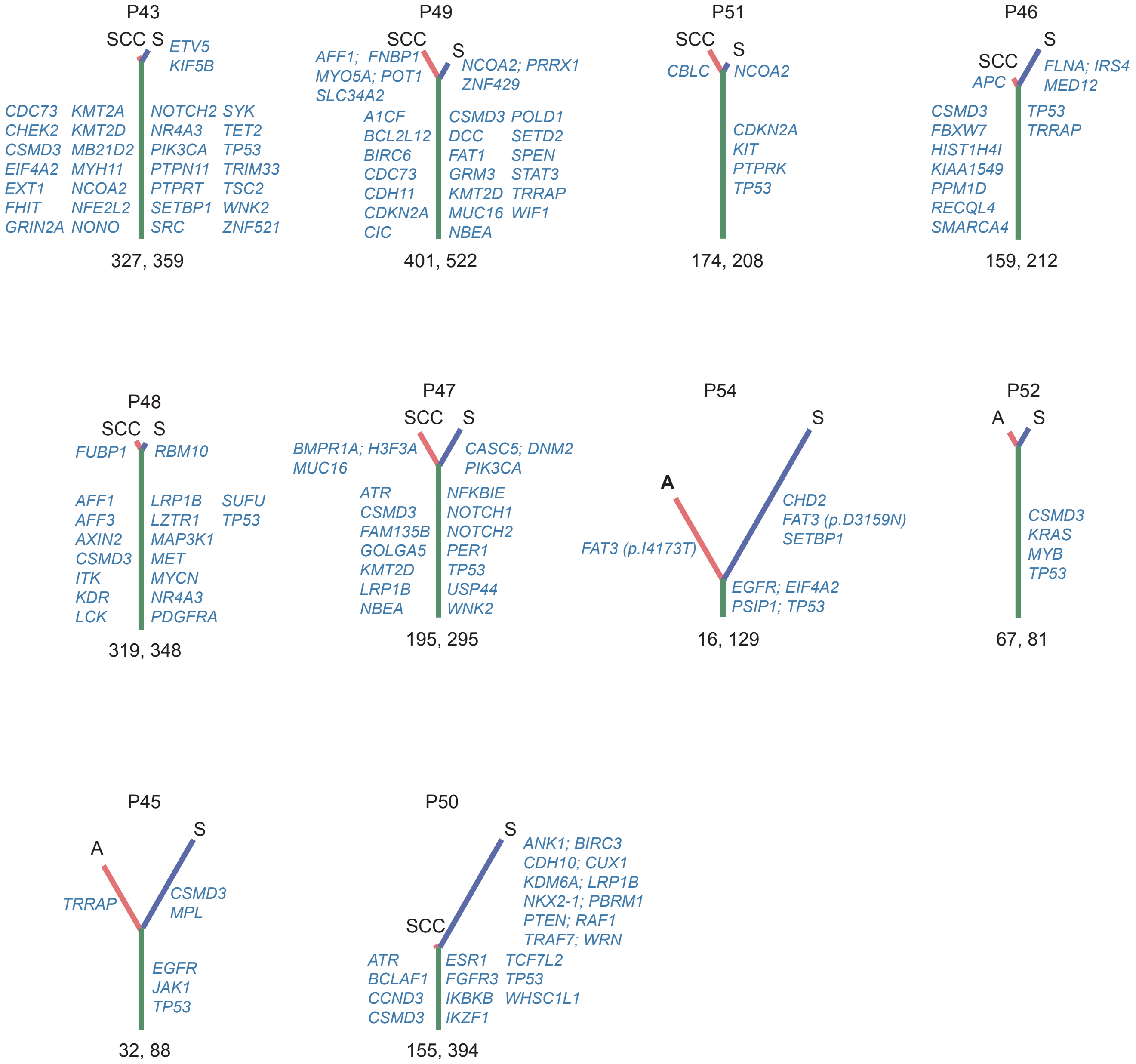

Supplementary Figure 5. Phylogenetic trees generated for the remaining 10 PSC samples. The length of the trunk (green) and branch (red or blue) represents the number of shared and specific nonsynonymous mutations respectively. Part of driver mutations is marked. The number of truncal and total nonsynonymous mutations is indicated below. SCC, squamous cell carcinoma component; A, adenocarcinoma component; S, sarcomatoid component.

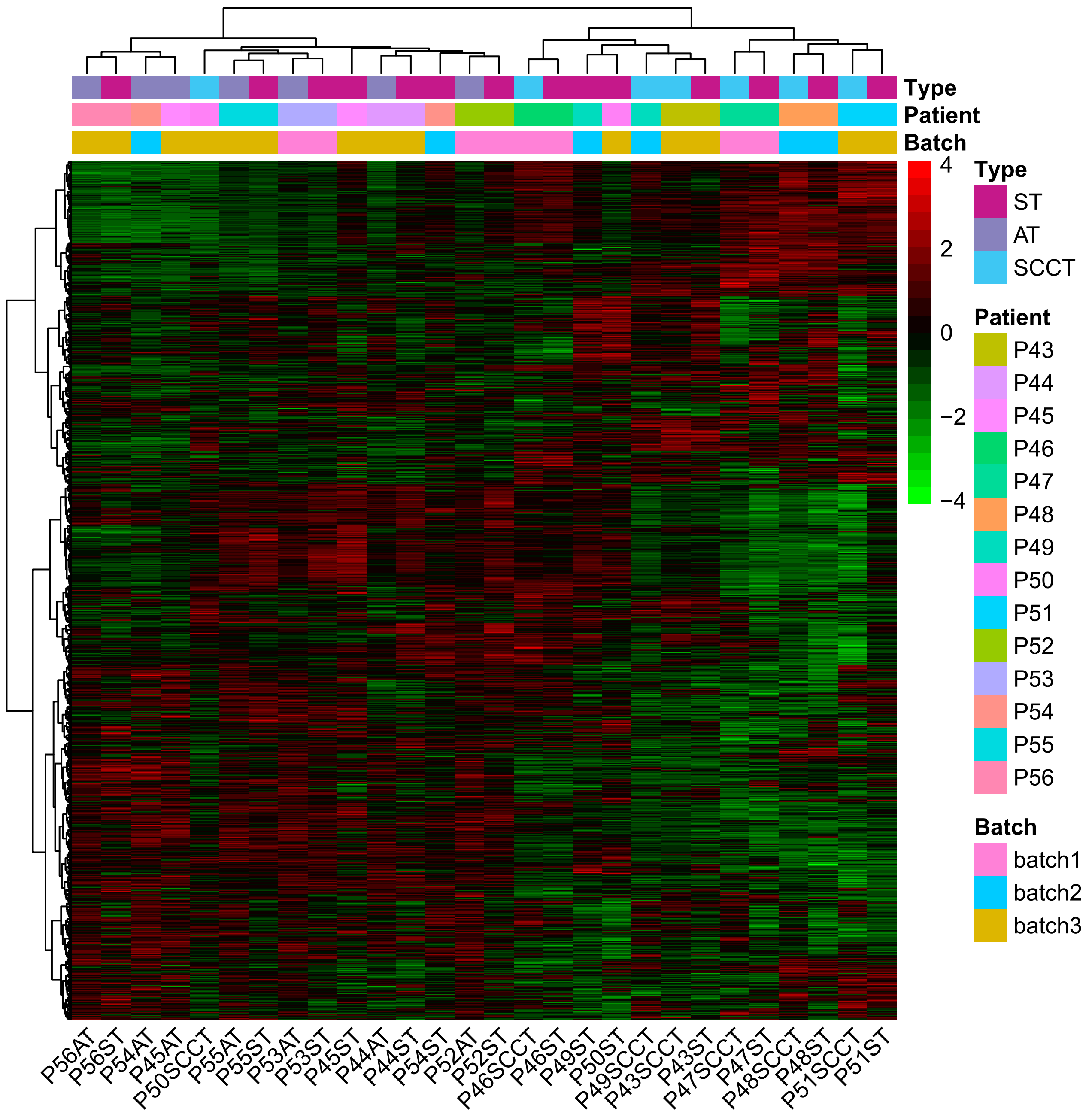

Supplementary Figure 6. The heatmap of unsupervised hierarchical clustering with transcriptome data from the epithelial and sarcomatoid components, annotated for the histological type, the patients and the batch. ST, sarcomatoid component; AT, adenocarcinoma component; SCCT, squamous cell carcinoma component. Source data are provided as a Source Data file.

A

	Differentially expressed genes	DMP-containing genes
KEGG Pathway	ECM-receptor interaction<br>Tight junction<br>PI3K-Akt signaling pathway<br>Focal adhesion<br>Cell adhesion molecules (CAMs)	Adherens junction<br>MAPK signaling pathway<br>VEGF signaling pathway<br>PI3K-Akt signaling pathway<br>Focal adhesion
Gene Ontology	Cell-cell junction<br>Extracellular matrix organization<br>Cell junction organization<br>Cell adhesion<br>Cell-cell junction organization	Adherens junction<br>Cell junction<br>Regulation of cell-matrix adhesion<br>Focal adhesion<br>Positive regulation of cell differentiation

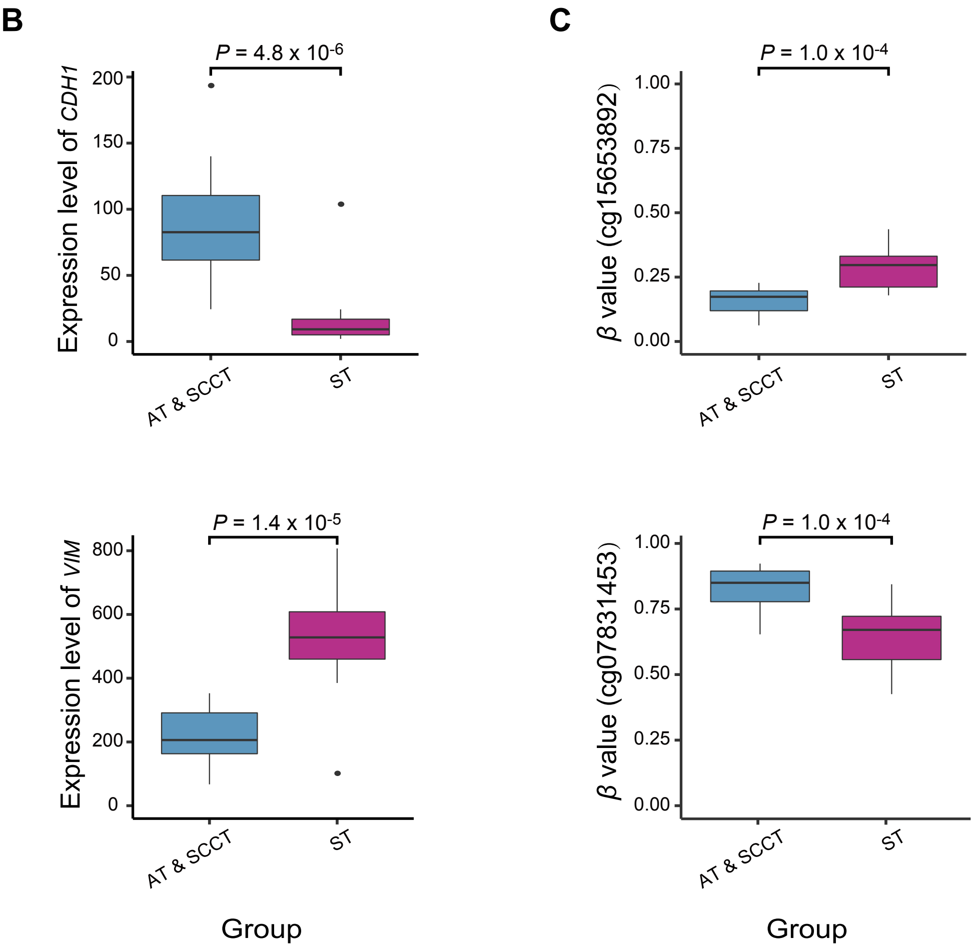

Supplementary Figure 7. The differences of transcriptional and DNA methylation profiles between the epithelial and sarcomatoid components. (A) Significant enrichment of differentially expressed genes and DMP-containing genes between the epithelial and sarcomatoid components in KEGG pathways and Gene Ontology terms. (B) The expression levels of *CDH1* and *VIM* are plotted for adenocarcinoma (AT) & squamous cell carcinoma (SCCT) components and sarcomatoid (ST) components as boxplots. (C) Boxplots show the β values of the probes in the promoter region (*CDH1*: cg15653892; *VIM*: cg07831453) of the two genes for the AT&SCCT and ST components. Center line, median; box limits, upper and lower quartiles; whiskers, 1.5× interquartile range; points, outliers. Two-sided Wilcoxon rank sum test was used for statistical analysis of (B) and (C). No *P*-value adjustment was applied. n = 14, 14 for AT&SCCT and ST group, respectively. *CDH1*, cadherin 1; *VIM*, vimentin.

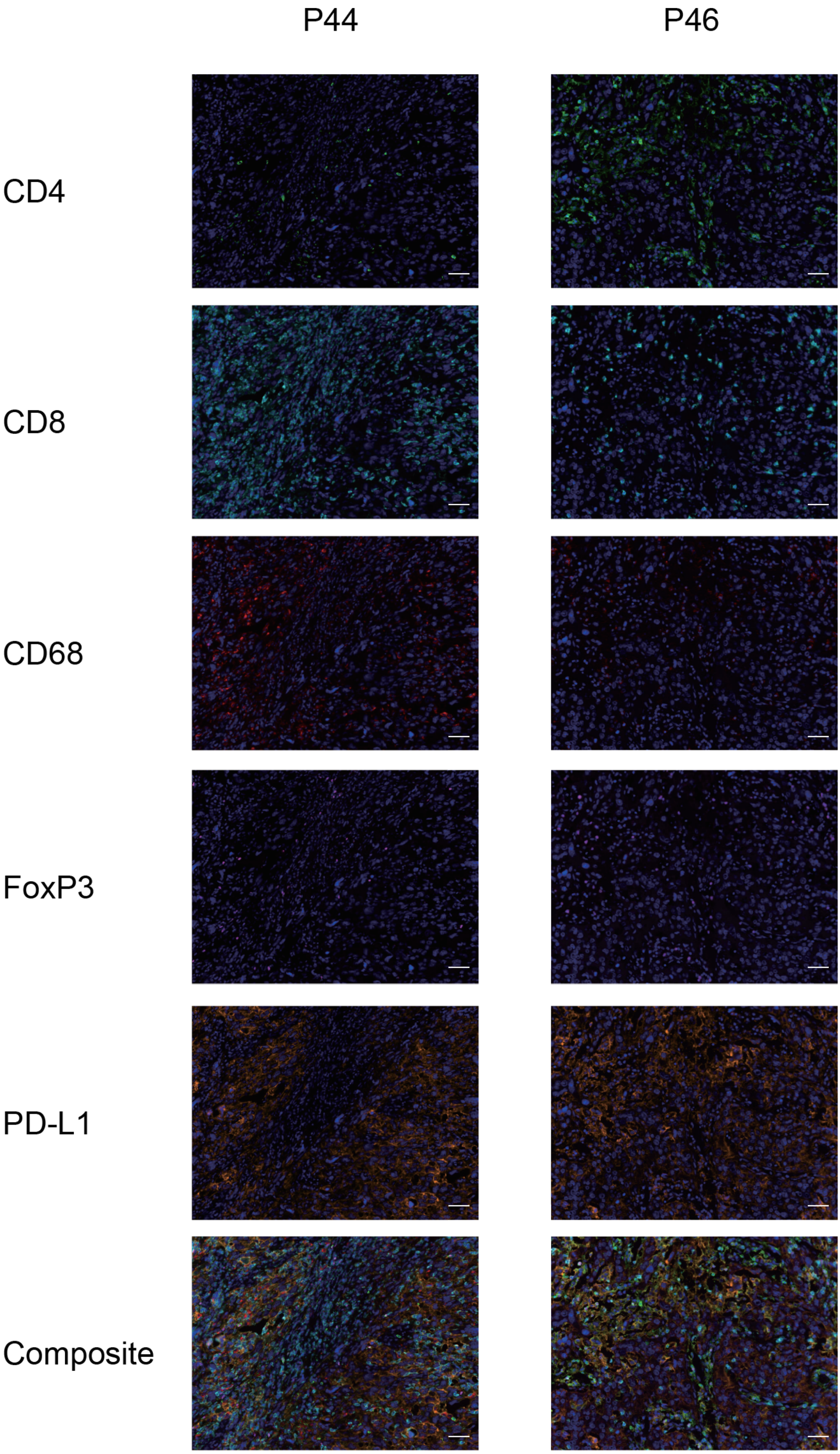

Supplementary Figure 8. Representative images of fluorescent multiplex immunohistochemical analysis of CD4, CD8, CD68, FoxP3 and PD-L1. Scale bar: 50μm. The fluorescent multiplex immunohistochemical analysis was performed on the epithelial and sarcomatoid components of 14 patients. CD4, cluster of differentiation 4; CD8, cluster of differentiation 8; CD68, cluster of differentiation 68; FoxP3, forkhead box P3; PD-L1, programmed death ligand 1.

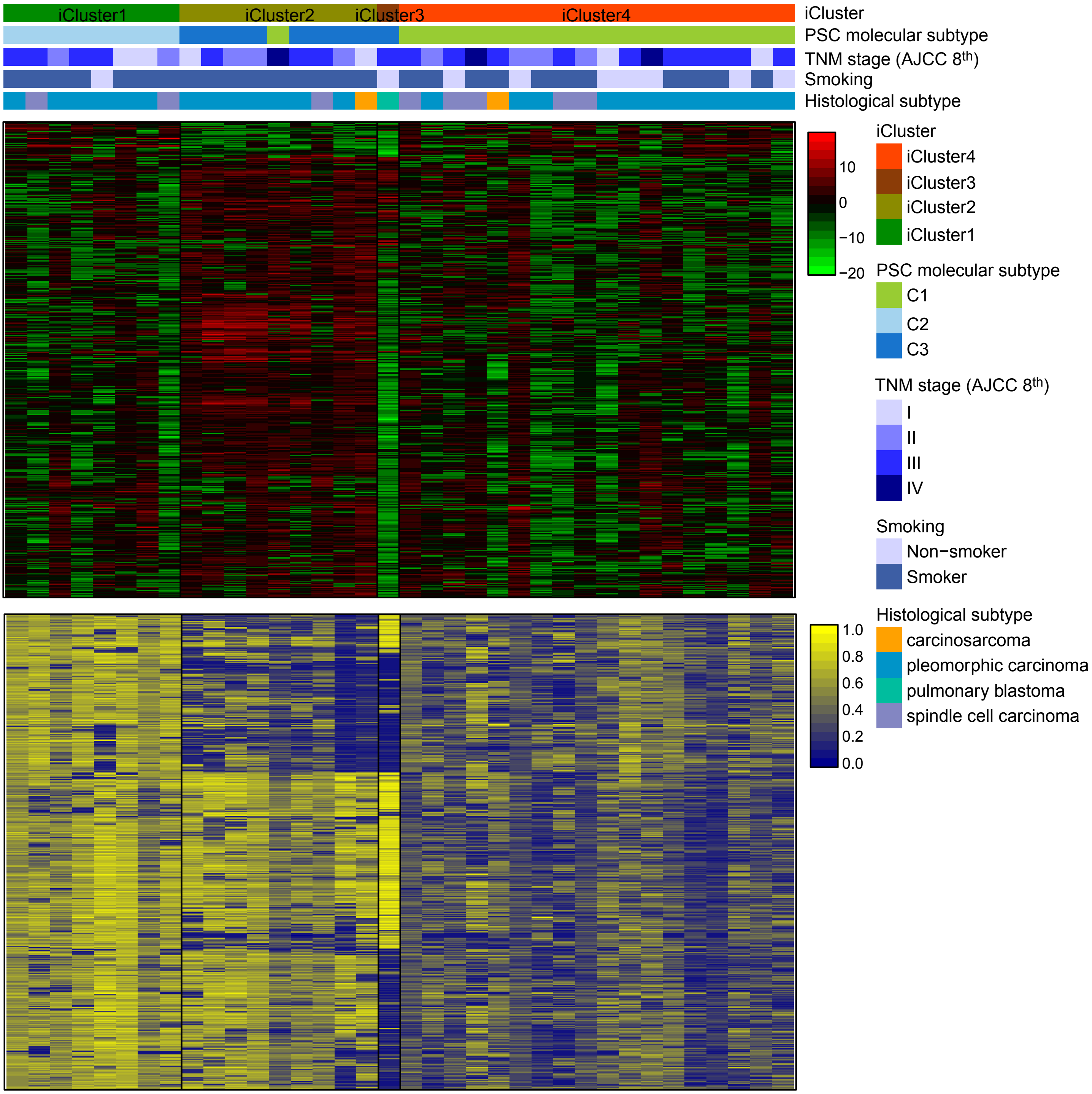

Supplementary Figure 9. Unsupervised iCluster analysis, which integrates transcriptome and DNA methylation data. PSC tumors are classified into four iCluster subtypes, annotated for the molecular subtype of PSC, TNM stage, smoking status and histological subtype. The profiles of transcriptome and DNA methylation are displayed in the middle and bottom panel, respectively.

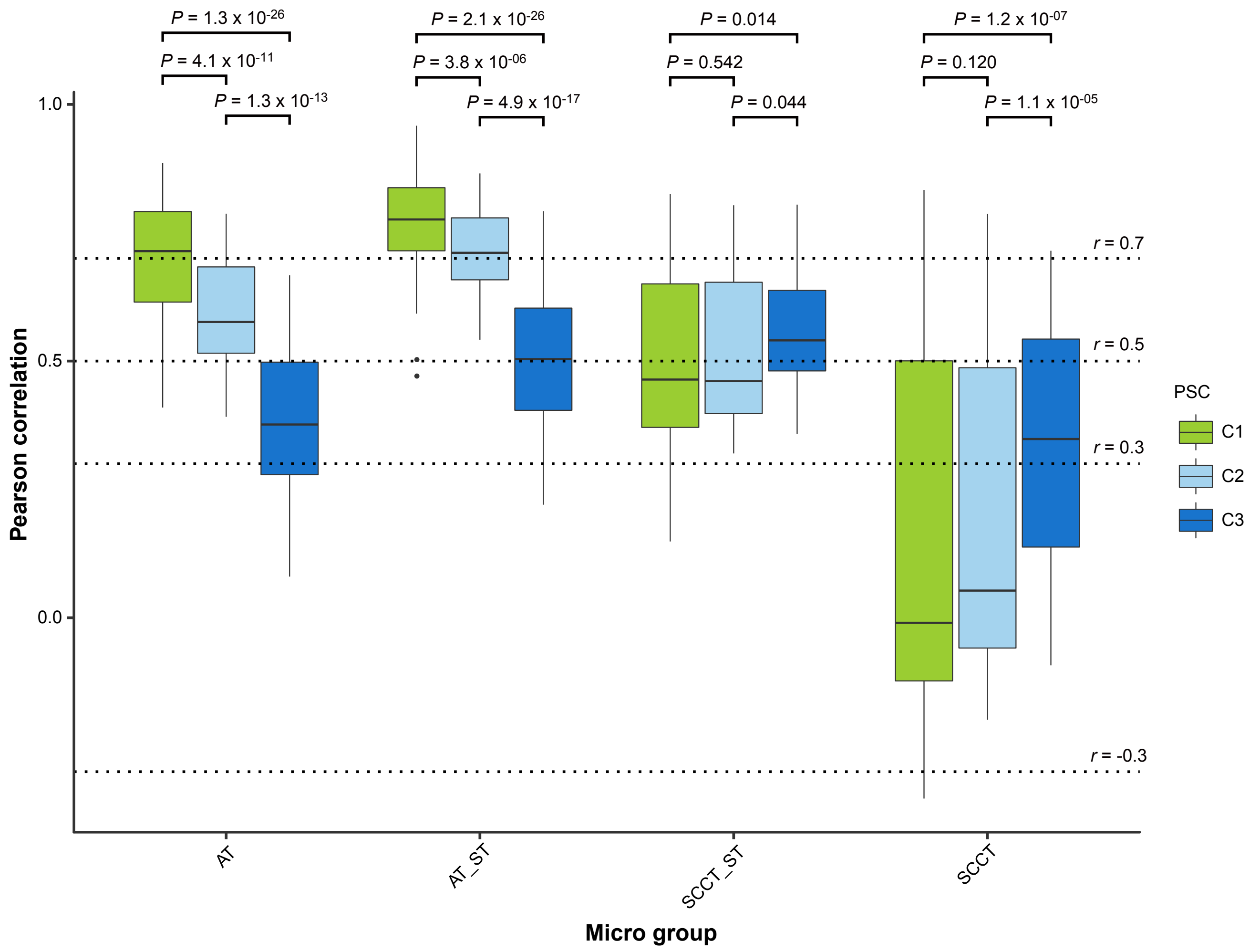

Supplementary Figure 10. Pearson correlations of DNA methylation profiles between pairs from subclassified samples (C1, C2 and C3) and microdissected samples (adenocarcinoma (AT) and corresponding sarcomatoid components (AT_ST), squamous cell carcinoma (SCCT) and corresponding sarcomatoid components (SCCT_ST)) are plotted as boxplots. Center line, median; box limits, upper and lower quartiles; whiskers, 1.5× interquartile range; points, outliers. Source data are provided as a Source Data file.

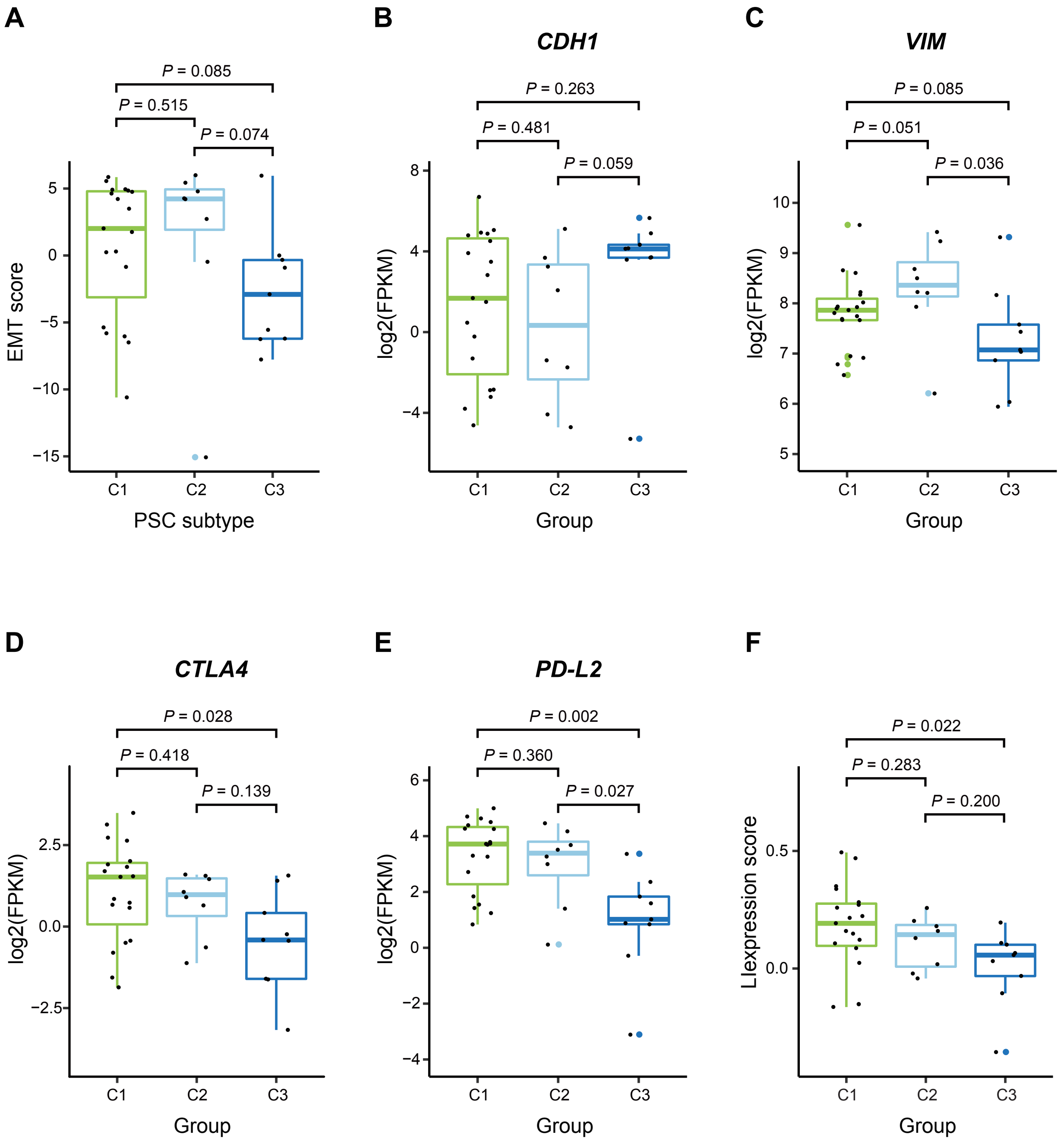

Supplementary Figure 11. Boxplots show the EMT scores (A), the expression levels of *CDH1* (B), *VIM* (C), *CTLA4* (D) and *PD-L2* (E) and the LIexpression scores (F) for C1, C2 and C3. Center line, median; box limits, upper and lower quartiles; whiskers, 1.5× interquartile range; colored points, outliers. Two-sided Wilcoxon rank sum test was used for statistical analysis. No *P*-value adjustment was applied. n = 19, 8, 9 for C1, C2 and C3, respectively. *CDH1*, cadherin 1; *VIM*, vimentin; *CTLA4*, cytotoxic T-lymphocyte associated protein 4; *PD-L2*, programmed death ligand 2.

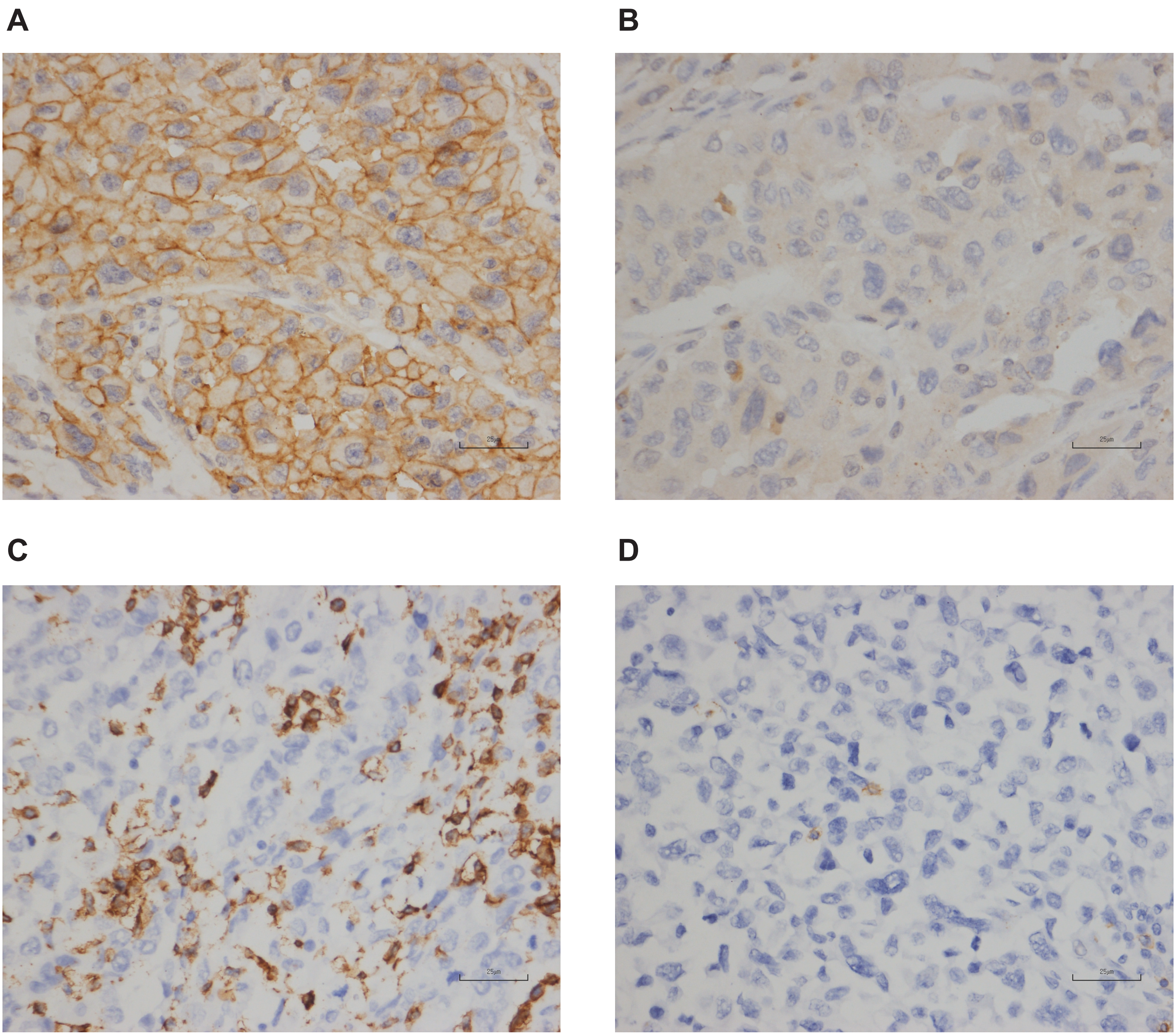

Supplementary Figure 12. Representative images of immunohistochemical staining for PD-L1 and CD8. (A) Positive staining for PD-L1. (B) Negative staining for PD-L1. (C) High density of CD8-positive lymphocyte infiltration. (D) Low density of CD8-positive lymphocyte infiltration. Scale bar: 25μm. 56 biologically independent PSC samples were stained for PD-L1 and CD8. PD-L1, programmed death ligand 1; CD8, cluster of differentiation 8.

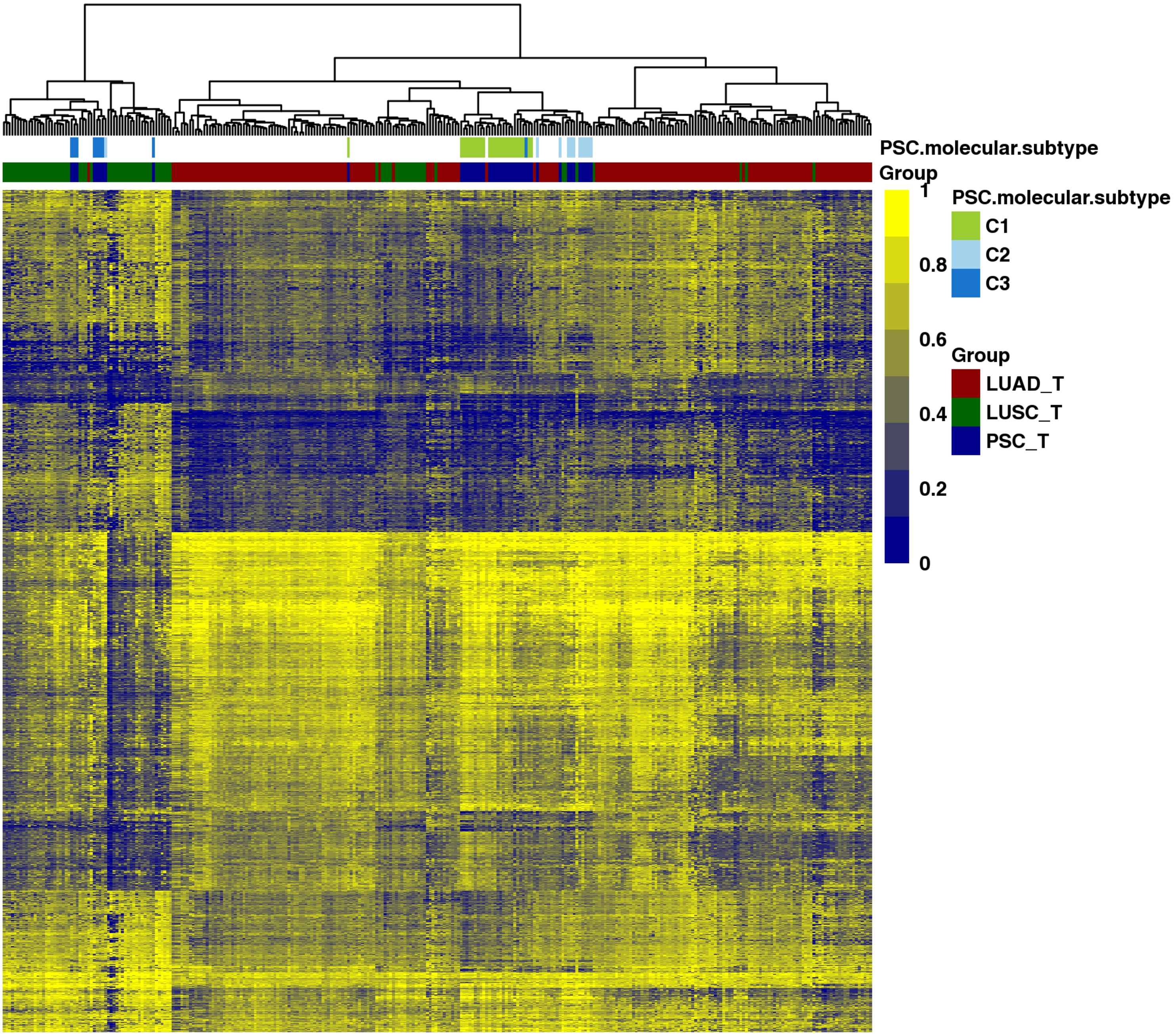

Supplementary Figure 13. Unsupervised hierarchical clustering of DNA methylation data of PSC and TCGA LUSC and LUAD. The analysis yields two major clusters, dominant by LUSC and LUAD respectively.

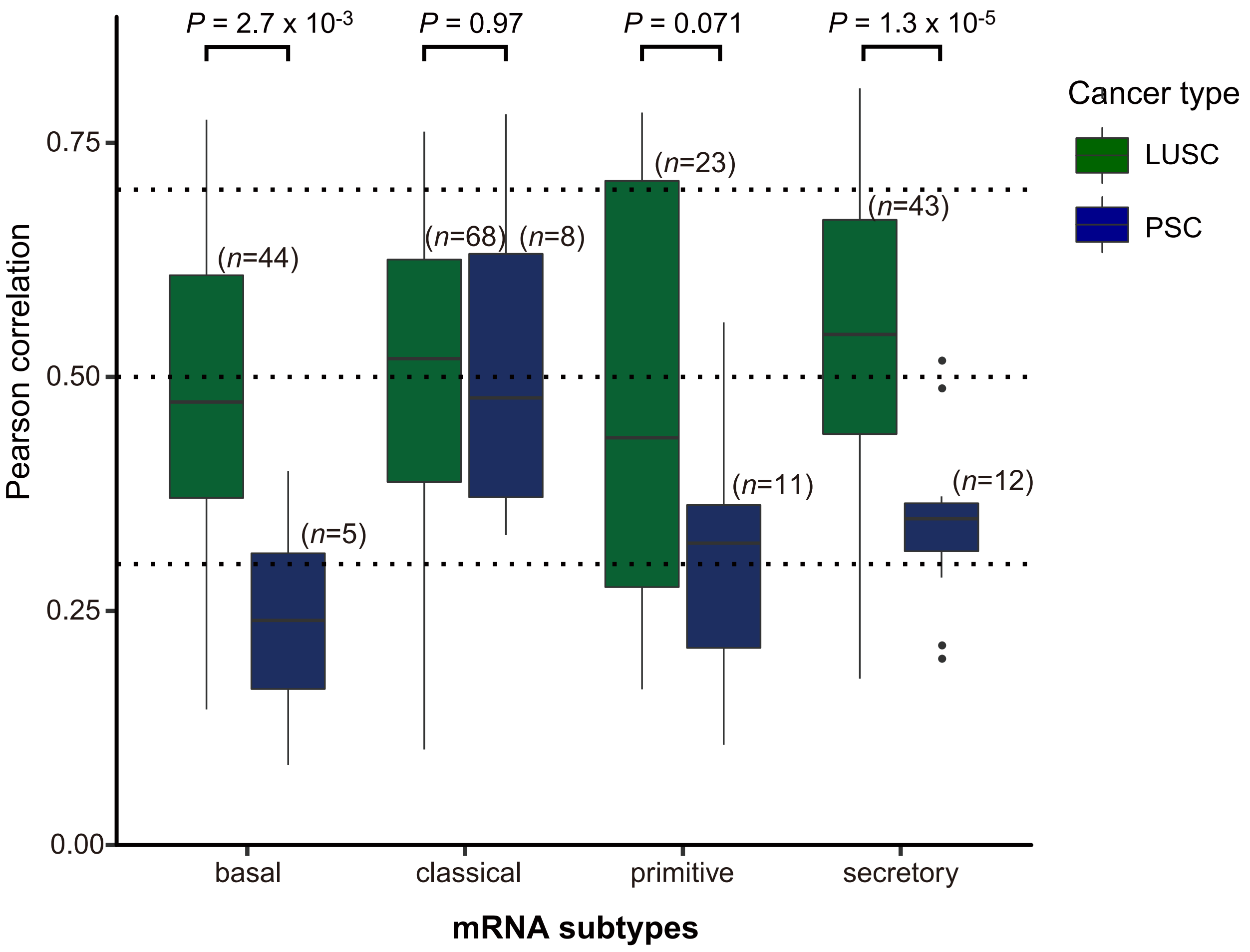

Supplementary Figure 14. Pearson correlations of expression profiles between PSC samples or TCGA LUSC samples and the predictor centroids for the four LUSC transcriptional subtypes are plotted as boxplots. Center line, median; box limits, upper and lower quartiles; whiskers, 1.5× interquartile range; points, outliers. Source data are provided as a Source Data file.

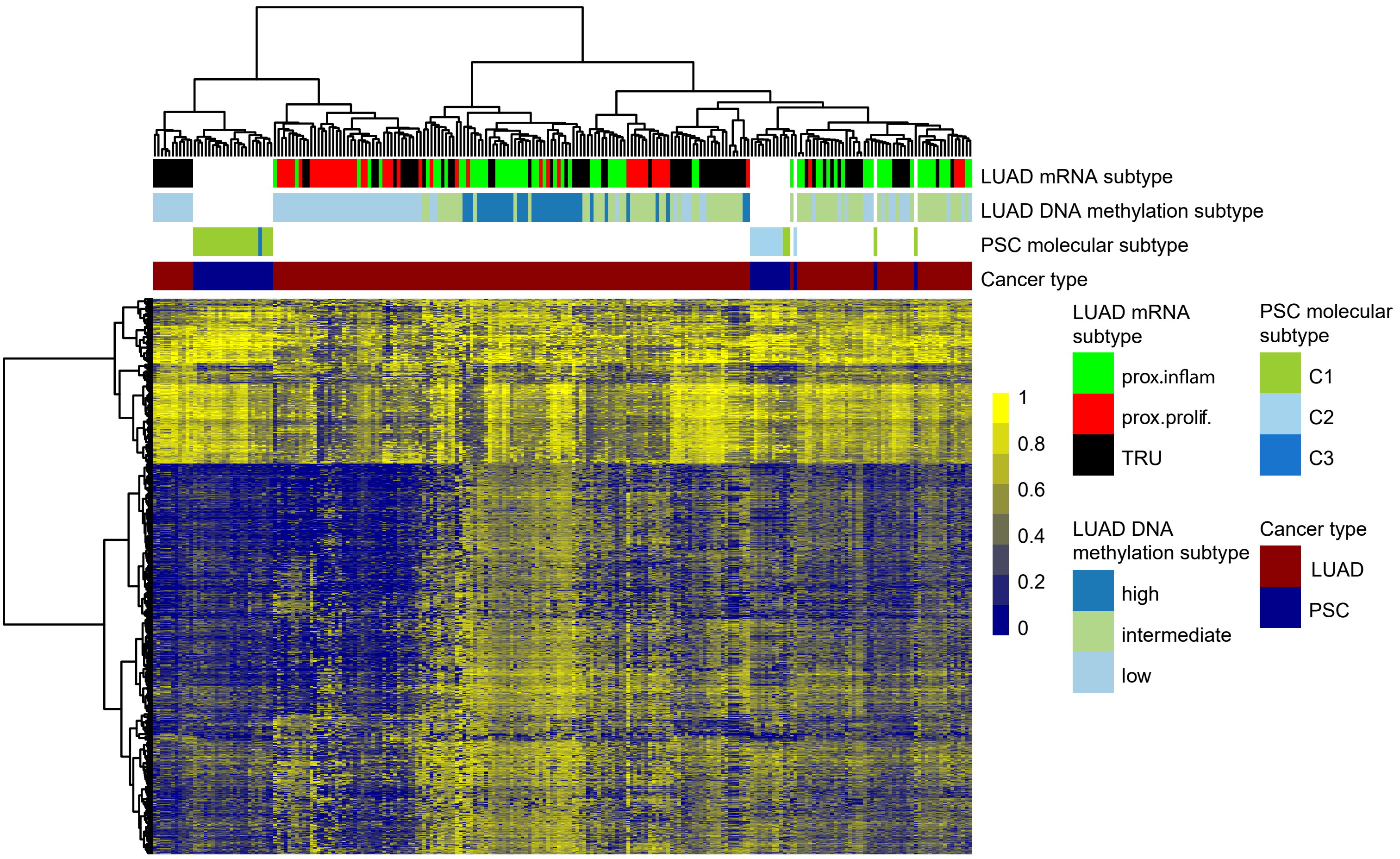

Supplementary Figure 15. Unsupervised hierarchical clustering of DNA methylation data of PSC_AD and TCGA LUAD. PSC_AD tumors are divided into two subsets, dominant by samples in C1 and C2, respectively.

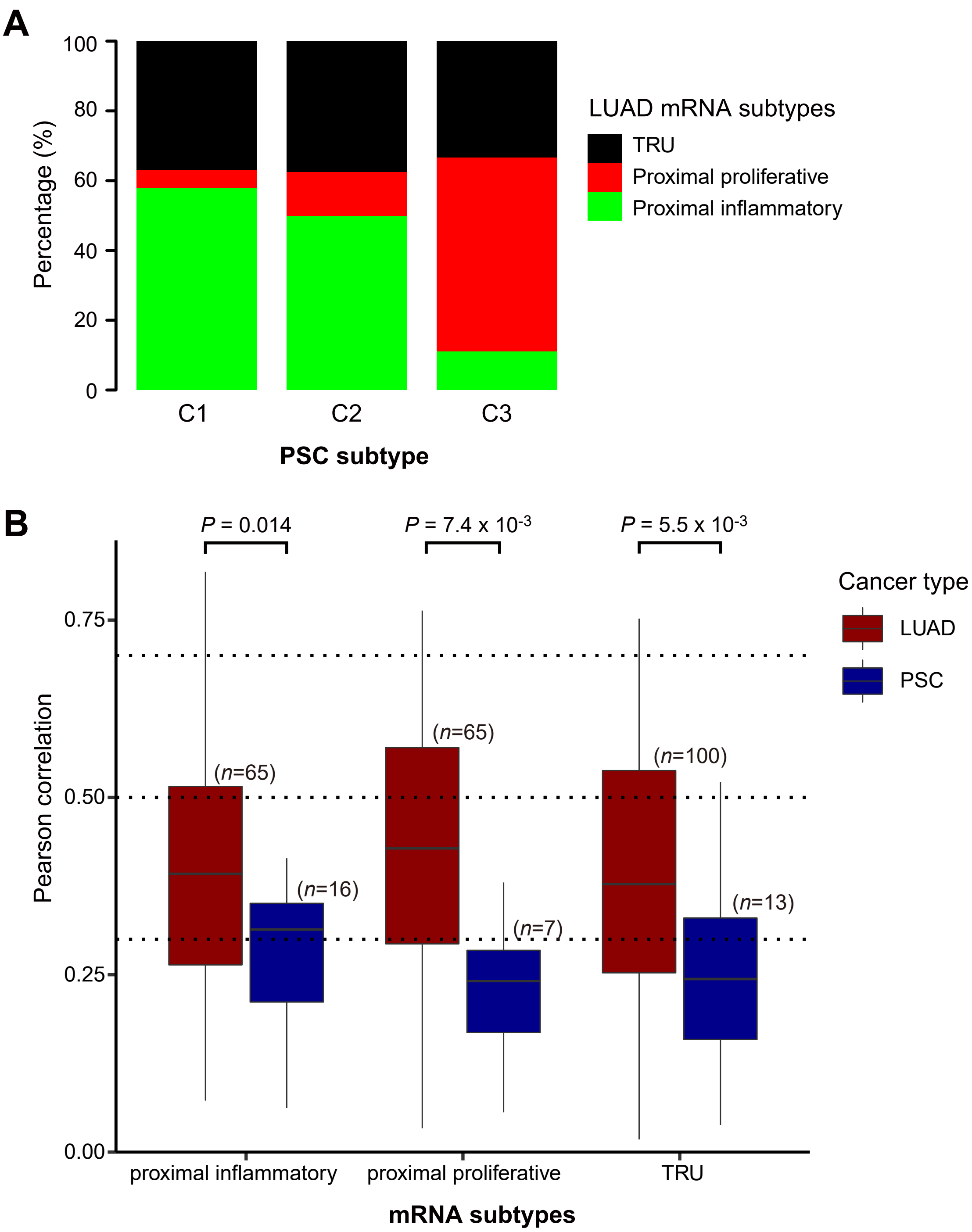

Supplementary Figure 16. LUAD transcriptional subtype prediction for PSC. (A) The proportion of the three LUAD transcriptional subtypes in three clusters of PSC. (B) Pearson correlations of expression profiles between PSC samples or TCGA LUAD samples and the predictor centroids for the three LUAD transcriptional subtypes are plotted as boxplots. Center line, median; box limits, upper and lower quartiles; whiskers, 1.5× interquartile range; points, outliers. Source data are provided as a Source Data file.
