# Supplementary material for: Integrated molecular characterization reveals potential therapeutic strategies for pulmonary sarcomatoid carcinoma
Source: Nat Commun. 2020 Sep 28;11:4878. doi: 10.1038/s41467-020-18702-3 (PMC7522294; doi:10.1038/s41467-020-18702-3)
Supplement: Supplementary file 2 — Description of Additional Supplementary Files [file 41467_2020_18702_MOESM2_ESM.docx]

**File Name: Supplementary Data 1**

**Description:** Sample Overview

**File Name: Supplementary Data 2**

**Description:** Information on the Sequencing Quality

**File Name: Supplementary Data 3**

**Description:** List of Somatic Nonsynonymous Mutations

**File Name: Supplementary Data 4**

**Description:** Significantly Mutated Genes Identified by Four Softwares (MutSigCV, MuSiC, OncodriveCLUST, Oncodrive-FM)

**File Name: Supplementary Data 5**

**Description:** Recurrent Copy Number Alterations Identified by GISTIC Analysis

**File Name: Supplementary Data 6**

**Description:** Pan-cancer Analysis of PSC and TCGA Tumor Types

**File Name: Supplementary Data 7**

**Description:** Molecular Characteristics Related to the Molecular Classification of PSC
