# Supplementary material for: Integrated molecular characterization reveals potential therapeutic strategies for pulmonary sarcomatoid carcinoma
Source: Nat Commun. 2020 Sep 28;11:4878. doi: 10.1038/s41467-020-18702-3 (PMC7522294; doi:10.1038/s41467-020-18702-3)
Supplement: Supplementary file 10 — Reporting Summary [file 41467_2020_18702_MOESM10_ESM.pdf]

nature research

Corresponding author(s): Jie He

Last updated by author(s): Jul 24, 2020

Reporting Summary

Nature Research wishes to improve the reproducibility of the work that we publish. This form provides structure for consistency and transparency in reporting. For further information on Nature Research policies, see Authors & Referees and the Editorial Policy Checklist .

Statistics

For all statistical analyses, confirm that the following items are present in the figure legend, table legend, main text, or Methods section.

n/a	Confirmed	
☐	☒	The exact sample size (*n*) for each experimental group/condition, given as a discrete number and unit of measurement
☐	☒	A statement on whether measurements were taken from distinct samples or whether the same sample was measured repeatedly
☐	☒	The statistical test(s) used AND whether they are one- or two-sided<br>*Only common tests should be described solely by name; describe more complex techniques in the Methods section.*
☐	☒	A description of all covariates tested
☐	☒	A description of any assumptions or corrections, such as tests of normality and adjustment for multiple comparisons
☐	☒	A full description of the statistical parameters including central tendency (e.g. means) or other basic estimates (e.g. regression coefficient) AND variation (e.g. standard deviation) or associated estimates of uncertainty (e.g. confidence intervals)
☐	☒	For null hypothesis testing, the test statistic (e.g. *F*, *t*, *r*) with confidence intervals, effect sizes, degrees of freedom and *P* value noted<br>*Give P values as exact values whenever suitable.*
☒	☐	For Bayesian analysis, information on the choice of priors and Markov chain Monte Carlo settings
☐	☒	For hierarchical and complex designs, identification of the appropriate level for tests and full reporting of outcomes
☐	☒	Estimates of effect sizes (e.g. Cohen's *d*, Pearson's *r*), indicating how they were calculated

Our web collection on statistics for biologists contains articles on many of the points above.

Software and code

Policy information about availability of computer code

Data collection	No specific software was used.
Data analysis	Softwares used in this study are publically available and stated in the Methods section where applicable.<br>Whole exome sequencing analysis: Trimmomatic 0.33, BWA-MEM v0.7.10-r789, MuTect v3.1-0-g72492bb, Strelka v1.0.14, Integrative Genomics Viewer v2.3.34, Control-FREEC v10.5, GISTIC 2.0, R package deconstructSigs v1.8.0, MutSigCV v1.41, MuSiC v0.4, OncodriveCLUST v0.4.1, Oncodrive-FM v1.0.3, R package ROCR v1.0-7; RNA sequencing analysis: Trimmomatic 0.33, HISAT2 v2.0.5, SAMtools v1.3, BCFtools v1.3.1, StringTie v1.3.1c, R package Ballgown v2.8.4, Cyber-T v1, KOBAS v3.0, R package GSVA v1.30.0, R package FactoMineR v1.42; DNA methylation data processing: R package ChAMP v2.9.10, R package limma v3.34.9; Integrative clustering: R package iClusterPlus v1.16.0; Fluorescent multiplex IHC: inForm v2.3.0; Statistics analysis: R v3.2.0.

For manuscripts utilizing custom algorithms or software that are central to the research but not yet described in published literature, software must be made available to editors/reviewers. We strongly encourage code deposition in a community repository (e.g. GitHub). See the Nature Research guidelines for submitting code & software for further information.

Data

Policy information about availability of data

All manuscripts must include a data availability statement. This statement should provide the following information, where applicable:

- Accession codes, unique identifiers, or web links for publicly available datasets
- A list of figures that have associated raw data
- A description of any restrictions on data availability

WES and RNA-seq data have been deposited at The National Omics Data Encyclopedia (NODE) under accession number OEP000661 [https://www.biosino.org/node/project/detail/OEP000661]. DNA methylation data have been deposited at NODE under accession number OEP001064 [https://www.biosino.org/node/project/detail/OEP001064]. The clinical data are provided in Supplementary Data 1. A complete list of somatic nonsynonymous mutations can be found in Supplementary Data 3. The data supporting Fig. 2A, 2B, 4A, 4B, 5A, 5C and 5D and Supplementary Figure 3, 7B and 11 of the study are available in the Supplementary Data files. The

source data underlying Fig. 1B, 2C, 3A, 3B, 3D, 4C, 6B, 6C and 6D and Supplementary Figure 6, 10, 14 and 16 are provided as a Source Data file. The DNA methylation data of PBMCs were obtained from GEO dataset with accession number: GSE35069. The transcriptome and DNA methylation data of TCGA LUAD and LUSC were collected from the following web-links https://portal.gdc.cancer.gov/projects/TCGA-LUAD and https://portal.gdc.cancer.gov/projects/TCGA-LUSC, respectively. All the other relevant data of the study are available from the corresponding authors upon reasonable request.

Field-specific reporting

Please select the one below that is the best fit for your research. If you are not sure, read the appropriate sections before making your selection.

[x] Life sciences [ ] Behavioural & social sciences [ ] Ecological, evolutionary & environmental sciences

For a reference copy of the document with all sections, see nature.com/documents/nr-reporting-summary-flat.pdf

Life sciences study design

All studies must disclose on these points even when the disclosure is negative.

Sample size

No statistical methods were used to determine the sample size. As pulmonary sarcomatoid carcinoma is a rare subtype of lung cancer, the sample size was determined by the access and availability of patient samples and materials.

Data exclusions

No data were excluded from the analyses.

Replication

The somatic mutations of TP53, EGFR, KRAS, PIK3CA, MET, PTEN, BRAF and NF1 detected by whole exome sequencing (WES) were validated by PCR-Sanger sequencing and/or RNA-seq. We performed targeted sequencing (average sequencing depth: 3229×) and checked whether a specific mutation was exclusively identified in only one component of the same tumor. By this procedure, 85% (40/47) of the covered specific mutations were confirmed as truly specific mutations. For 3 of the rest specific mutations, the allele frequencies were much lower in the components, in which WES did not identify the specific mutations, but the tumor purity reviewed by pathologists was similar, indicating the true intratumoral heterogeneity. The other 4 mutations were excluded in the further analysis, as (1) the depth of targeted sequencing was extremely low (1 mutation); (2) the private mutation resulted from random sampling of sequencing reads (1 mutation); (3) the private mutations were not detected in targeted sequencing (2 mutations).

Randomization

Randomization and blinding were not applicable to this study, as the patients enrolled in this study were recruited retrospectively, and the clinical data were retrieved from digital medical records.

Blinding

Randomization and blinding were not applicable to this study, as the patients enrolled in this study were recruited retrospectively, and the clinical data were retrieved from digital medical records.

Reporting for specific materials, systems and methods

We require information from authors about some types of materials, experimental systems and methods used in many studies. Here, indicate whether each material, system or method listed is relevant to your study. If you are not sure if a list item applies to your research, read the appropriate section before selecting a response.

Materials & experimental systems

n/a	Involved in the study	
[ ]	[x]	Antibodies
[x]	[ ]	Eukaryotic cell lines
[x]	[ ]	Palaeontology
[x]	[ ]	Animals and other organisms
[ ]	[x]	Human research participants
[x]	[ ]	Clinical data

Methods

n/a	Involved in the study	
[x]	[ ]	ChIP-seq
[x]	[ ]	Flow cytometry
[x]	[ ]	MRI-based neuroimaging

Antibodies

Antibodies used

anti-CD8 rabbit monoclonal antibody (D8A8Y, 1:1000, Cell Signaling Technology, Cat No. 85336); anti-PD-L1 rabbit monoclonal antibody (28-8, 1:500, Abcam, Cat No. ab205921); anti-PD-L1 rabbit monoclonal antibody (E1L3N, 1:100, Cell Signaling Technology, Cat No. 13684); anti-CD4 rabbit monocloncal antibody (EP204, 1:100, Zsbio, Cat No. ZA-0519); anti-CD68 mouse monoclonal antibody (KP1, 1:500, Zsbio, Cat No. ZM-0060); anti-CD8 rabbit monoclonal antibody (SP16, 1:100, Zsbio, Cat No. ZA-0508); anti-FoxP3 mouse monoclonal antibody (236A/E7, 1:100, Abcam, Cat No. ab20034).

Validation

CD8 antibody (D8A8Y) was validated by the immunohistochemical analysis of paraffin-embedded human Crohn's diseased colon, lung carcinoma and lymphoma. PD-L1, CD4, CD68 and CD8 antibodies (28-8, EP204, KP1, SP16) were validated by the immunohistochemical analyses of paraffin-embedded human tonsil. PD-L1 antibody (E1L3N) was validated by the immunohistochemical analysis of paraffin-embedded human non-small cell lung carcinoma and placenta. FoxP3 antibody (236A/E7) was validated by the immunohistochemical analysis of paraffin-embedded human tonsil and breast cancer.

Human research participants

Policy information about studies involving human research participants

Population characteristics	We enrolled 56 patients diagnosed with pulmonary sarcomatoid carcinoma who underwent surgery at Cancer Hospital, Chinese Academy of Medical Sciences. The median age of the patients was 62.5 years (range, 45-78 years). 42 (75.0%) of the patients were male, and 39 (69.6%) were smokers.
Recruitment	The study retrospectively collected paired tumor samples and normal lung tissues from 56 patients diagnosed with pulmonary sarcomatoid carcinoma (PSC) who underwent surgery at Cancer Hospital, Chinese Academy of Medical Sciences. Patients were selected only if they had no prior treatment before surgery and if they had tumor and paired normal lung tissue samples available. Because of the rarity of PSC, we enrolled as many patients as possible in this study. Therefore, no self-selection bias was present.
Ethics oversight	The Ethics Committee of National Cancer Center/Cancer Hospital, Chinese Academy of Medical Sciences and Peking Union Medical College approved the study protocol (18-224/1782).

Note that full information on the approval of the study protocol must also be provided in the manuscript.
